# Bi-allelic variants in *CELSR3* are implicated in central nervous system and urinary tract anomalies

**DOI:** 10.1038/s41525-024-00398-9

**Published:** 2024-03-01

**Authors:** Jil D. Stegmann, Jeshurun C. Kalanithy, Gabriel C. Dworschak, Nina Ishorst, Enrico Mingardo, Filipa M. Lopes, Yee Mang Ho, Phillip Grote, Tobias T. Lindenberg, Öznur Yilmaz, Khadija Channab, Steve Seltzsam, Shirlee Shril, Friedhelm Hildebrandt, Felix Boschann, André Heinen, Angad Jolly, Katherine Myers, Kim McBride, Mir Reza Bekheirnia, Nasim Bekheirnia, Marcello Scala, Manuela Morleo, Vincenzo Nigro, Annalaura Torella, TUDP consortium, Michele Pinelli, Valeria Capra, Andrea Accogli, Silvia Maitz, Alice Spano, Rory J. Olson, Eric W. Klee, Brendan C. Lanpher, Se Song Jang, Jong-Hee Chae, Philipp Steinbauer, Dietmar Rieder, Andreas R. Janecke, Julia Vodopiutz, Ida Vogel, Jenny Blechingberg, Jennifer L. Cohen, Kacie Riley, Victoria Klee, Laurence E. Walsh, Matthias Begemann, Miriam Elbracht, Thomas Eggermann, Arzu Stoppe, Kyra Stuurman, Marjon van Slegtenhorst, Tahsin Stefan Barakat, Maureen S. Mulhern, Tristan T. Sands, Cheryl Cytrynbaum, Rosanna Weksberg, Federica Isidori, Tommaso Pippucci, Giulia Severi, Francesca Montanari, Michael C. Kruer, Somayeh Bakhtiari, Hossein Darvish, Heiko Reutter, Gregor Hagelueken, Matthias Geyer, Adrian S. Woolf, Jennifer E. Posey, James R. Lupski, Benjamin Odermatt, Alina C. Hilger

**Affiliations:** 1 Institute of Human Genetics, Medical Faculty, University of Bonn, Bonn, 53127 Germany; 2 Institute of Anatomy and Cell Biology, Medical Faculty, University of Bonn, Bonn, 53115 Germany; 3 Institute of Neuroanatomy, Medical Faculty, University of Bonn, Bonn, 53115 Germany; 4 Department of Neuropediatrics, University Hospital Bonn, Bonn, 53127 Germany; 5 Division of Cell Matrix Biology and Regenerative Medicine, School of Biological Sciences, Faculty of Biology Medicine and Health, University of Manchester, Manchester, UK; 6 Georg-Speyer-Haus, Institute for Tumor Biology and Experimental Therapy, 60596 Frankfurt am Main, Germany; 7 Division of Nephrology, Department of Pediatrics, Boston Children’s Hospital, Harvard Medical School, Boston, MA USA; 8 Institute of Medical Genetics and Human Genetics, Charité Universitätsmedizin Berlin, corporate member of Freie Universität Berlin and Humboldt-Universität zu Berlin, Berlin, Germany; 9 Department of Pediatrics, Medizinische Fakultät Carl Gustav Carus, Technische Universität Dresden, Dresden, Germany; 10 Department of Molecular & Human Genetics, Baylor College of Medicine, Houston, TX 77030 USA; 11 Medical Scientist Training Program, Baylor College of Medicine, Houston, TX USA; 12 Center for Cardiovascular Research, Nationwide Children’s Hospital, Department of Pediatrics, Ohio State University, Columbus, OH USA; 13 Department of Pediatrics, Renal Service, Texas Children’s Hospital, Houston, TX 77030 USA; 14 Department of Pediatrics, Baylor College of Medicine, Houston, TX 77030 USA; 15 Department of Neurosciences, Rehabilitation, Ophthalmology, Genetics, Maternal and Child Health (DINOGMI), University of Genoa, 16132 Genoa, Italy; 16 U.O.C. Genetica Medica, IRCCS Istituto Giannina Gaslini, 16147 Genoa, Italy; 17 Medical Genetics, Department of Precision Medicine, Università degli Studi della Campania ‘Luigi Vanvitelli’, via Luigi De Crecchio 7, 80138 Naples, Italy; 18 Telethon Institute of Genetics and Medicine, Pozzuoli, Naples Italy; 19 Department of Molecular Medicine and Medical Biotechnologies, University Federico II, Naples, Italy; 20 Genomics and Clinical Genetics, IRCCS Gaslini, Genoa, Italy; 21 Division of Medical Genetics, Department of Specialized Medicine, McGill University, Montreal, QC Canada; 22 Department of Human Genetics, McGill University, Montreal, QC Canada; 23 Medical Genetics Service, Oncology Department of Southern Switzerland, Ente Ospedaliero Cantonale, Lugano, Switzerland; 24 MBBM Foundation, Monza, Italy; 25 Center for Individualized Medicine, Mayo Clinic, Rochester, MN USA; 26 Department of Clinical Genomics, Mayo Clinic, Rochester, MN USA; 27 Department of Quantitative Health Sciences, Mayo Clinic, Rochester, MN USA; 28 Department of Pediatrics, Seoul National University College of Medicine, Seoul, Republic of Korea; 29 Department of Genomics Medicine, Rare Disease Center, Seoul National University Hospital, Seoul, Republic of Korea; 30 Division of Neonatology, Pediatric Intensive Care and Neuropediatrics, Comprehensive Center for Pediatrics, Medical University of Vienna, Vienna, Austria; 31 Division of Bioinformatics, Medical University of Innsbruck, 6020 Innsbruck, Austria; 32 Department of Pediatrics I, Medical University of Innsbruck, 6020 Innsbruck, Austria; 33 Division of Human Genetics, Medical University of Innsbruck, 6020 Innsbruck, Austria; 34 Department of Pediatrics and Adolescent Medicine, Division of Pediatric Pulmonology, Allergology and Endocrinology, Comprehensive Center for Pediatrics, Medical University of Vienna, 1090 Vienna, Austria; 35 Department of Clinical Medicine, Aarhus University, Aarhus, Denmark; 36 Department of Clinical Genetics, Aarhus University Hospital, Aarhus, Denmark; 37 Division of Medical Genetics, Department of Pediatrics, Duke University, Durham, NC USA; 38 Department of Pediatrics, Duke University Medical Center, Durham, NC USA; 39 Pediatric Neurology, Riley Hospital for Children Indiana University Health, Indianapolis, IN USA; 40 Institute for Human Genetics and Genomic Medicine, Medical Faculty, RWTH Aachen University, Aachen, Germany; 41 Division of Neuropediatrics and Social Pediatrics, Department of Pediatrics, Medical Faculty, RWTH Aachen University, 52074 Aachen, Germany; 42 Department of Clinical Genetics, Erasmus MC University Medical Center, Rotterdam, The Netherlands; 43 Department of Neurology, Columbia University Vagelos College of Physicians and Surgeons, New York, NY USA; 44 Department of Pathology, Columbia University Vagelos College of Physicians and Surgeons, New York, NY USA; 45 Division of Child Neurology, Department of Neurology, Columbia University Vagelos College of Physicians and Surgeons and NewYork-Presbyterian Morgan Stanley Children’s Hospital, New York, NY USA; 46 Department of Pediatrics, Columbia University Vagelos College of Physicians and Surgeons and NewYork-Presbyterian Morgan Stanley Children’s Hospital, New York, NY USA; 47 Institute for Genomic Medicine, Columbia University Vagelos College of Physicians and Surgeons, New York, NY USA; 48 Department of Genetic Counselling, The Hospital for Sick Children, Toronto, ON M5G 1X8 Canada; 49 Department of Molecular Genetics, University of Toronto, Toronto, ON M5S 1A1 Canada; 50 Division of Clinical and Metabolic Genetics, The Hospital for Sick Children, Toronto, ON M5G 1X8 Canada; 51 U.O. Genetica Medica, IRCCS Azienda Ospedaliero-Universitaria di Bologna, Bologna, Italy; 52 Pediatric Movement Disorders Program, Division of Pediatric Neurology, Barrow Neurological Institute, Phoenix Children’s Hospital, Phoenix, AZ USA; 53 Departments of Child Health, Neurology, and Cellular & Molecular Medicine, and Program in Genetics, University of Arizona College of Medicine–Phoenix, Phoenix, AZ USA; 54 Neuroscience Research Center, Faculty of Medicine, Golestan University of Medical Sciences, Gorgan, Iran; 55 Division Neonatology and Pediatric Intensive Care, Department of Pediatric and Adolescent Medicine, Friedrich-Alexander University of Erlangen-Nürnberg, Erlangen, Germany; 56 Institute of Human Genetics, Friedrich-Alexander University of Erlangen-Nürnberg, Erlangen, Germany; 57 Institute of Structural Biology, University Hospital Bonn, University of Bonn, Venusberg-Campus 1, 53127 Bonn, Germany; 58 Royal Manchester Children’s Hospital, Manchester University NHS Foundation Trust, Manchester Academic Health Science Centre, Manchester, UK; 59 Human Genome Sequencing Center, Baylor College of Medicine, Houston, TX 77030 USA; 60 Texas Children’s Hospital, Houston, TX 77030 USA; 61 Department of Pediatric and Adolescent Medicine, Friedrich-Alexander University of Erlangen-Nürnberg, Erlangen, 91054 Germany; 62 Research Center On Rare Kidney Diseases (RECORD), University Hospital Erlangen, 91054 Erlangen, Germany

**Keywords:** Development, Neurodevelopmental disorders, Kidney diseases, Genetic testing, Urogenital diseases

## Abstract

*CELSR3* codes for a planar cell polarity protein. We describe twelve affected individuals from eleven independent families with bi-allelic variants in *CELSR3*. Affected individuals presented with an overlapping phenotypic spectrum comprising central nervous system (CNS) anomalies (7/12), combined CNS anomalies and congenital anomalies of the kidneys and urinary tract (CAKUT) (3/12) and CAKUT only (2/12). Computational simulation of the 3D protein structure suggests the position of the identified variants to be implicated in penetrance and phenotype expression. CELSR3 immunolocalization in human embryonic urinary tract and transient suppression and rescue experiments of Celsr3 in fluorescent zebrafish reporter lines further support an embryonic role of *CELSR3* in CNS and urinary tract formation.

## Introduction

Co-occurrence of congenital anatomical and functional anomalies of the central nervous system (CNS) and congenital anomalies of the kidneys and urinary tract (CAKUT) have been previously reported, e.g. Galloway-Mowat syndrome [MIM 251300]^1^; CAKUTHED [MIM 617641]^2^; or NECRC [MIM 619522]^3^. The cadherin EGF LAG seven-pass G-type receptors (CELSRs) are as a subgroup of adhesion G protein-coupled receptors (aGPCRs) involved in many biological processes such as regulation of planar cell polarity (PCP) during embryonic development, neuronal and endocrine cell differentiation, vessel formation and axon guidance^4,5^. In the context of kidney development and pathophysiology, aGPCRs and in particular Celsr1 are known to play an important role in ureteric bud branching^6^. All CELSR family members CELSR1-3 have large ecto-domains for homophilic interactions followed by seven transmembrane segments and a cytoplasmic domain^4^. Expression studies of all three CELSR paralogs in xenopus and mice show distinct co-expression in the embryonic CNS and the pronephric system, a vertebrate kidney precursor^6–8^. *Celsr3* mutant mice show severe thalamocortical disconnection, decreased rubrospinal axons, corticospinal axons, spinal motoneurons and neuromuscular junctions, due to failure in axon guidance or outgrowth^9–11^. Rare monoallelic variants in human *CELSR3* have been associated with neural tube defects (NTDs)^4,12^, febrile seizures^13^ and Tourette disorder^14^.

Previously, we detected compound heterozygous variant alleles in *CELSR3* in an affected female with CAKUT and tethered cord syndrome^15^. Here, we report a total of twelve individuals with rare or novel bi-allelic variants in *CELSR3*. The affected individuals share an overlapping phenotypic spectrum comprising CNS anomalies, co-occurring CNS anomalies combined with CAKUT and CAKUT only. Computational simulation of the 3D protein structure suggests the position of the identified variants to be implicated in phenotype expression. Immuno-detection of CELSR3 in human embryonic urinary tract and transient suppression and rescue experiments of Celsr3 in fluorescent zebrafish reporter lines suggest an embryonic involvement in CNS and urinary tract formation.

## Results

### Individuals with bi-allelic variants in *CELSR3* present within a phenotypic spectrum

**Fig. 1:**
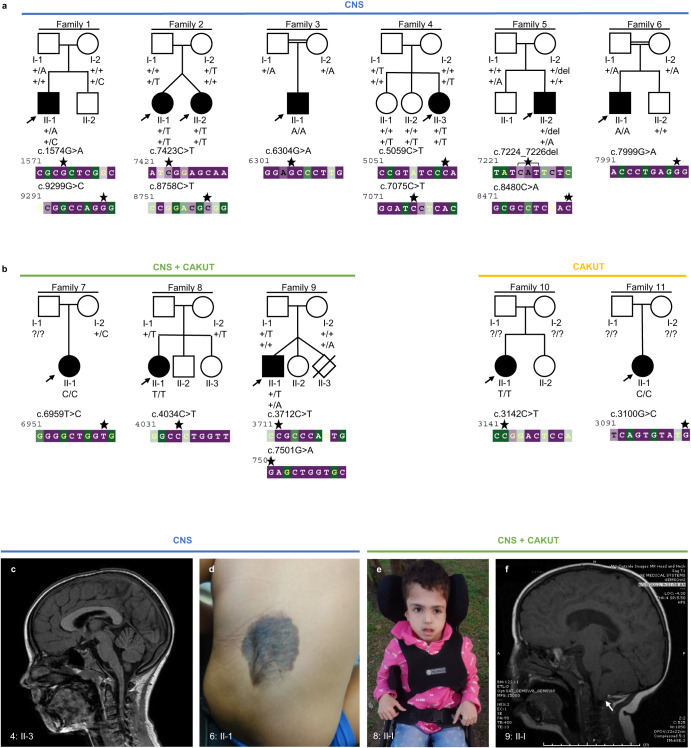
Families with bi-allelic variants in *CELSR3* and clinical images. **a** Pedigrees of six families (1–6) with a predominant central nervous system (CNS) phenotype. **b** Pedigrees of three families (7–9) with combined CNS phenotype and congenital anomalies of the kidneys and urinary tract (CAKUT), and two families (10, 11) with CAKUT only. The evolutionary conservation of the affected sequence (bp) was estimated with the ConSurf server from variable (green) to conserved (purple). Asterisks: Position of the respective variants. The arrows indicate probands. Filled shapes should reflect affected status. **c** Brain magnetic resonance image (MRI) of 4: II-3 showing pachygyria. **d** Photograph of 6: II-1 showing a congenital hairy melanocytic nevus with a diameter of 0.1 to 0.15 meter at the level of the lower lumbal spine. Radiologic imaging of the spine was not performed here. **e** Photograph of 8: II-1 showing macrocephaly, high and prominent forehead and very small and low-set ears. **f** MRI of 9: II-1. Arrow: Chiari malformation type 1 (cerebellar tonsillar herniation).

Six of the twelve described individuals presented with homozygous missense and five with compound heterozygous missense *CELSR3* variant alleles (Table 1). Individual 5: II-2 carried a heterozygous missense variant and an in-frame-deletion in trans. Seven of twelve individuals presented with a predominant CNS phenotype (1: II-1, 2: II-1, 2: II-2, 3: II-1, 4: II-3, 5: II-2, 6: II-1), three presented with a combined CNS and CAKUT phenotype (7: II-1, 8: II-1, 9: II-1) and two presented with CAKUT only (10: II-1, 11: II-1) (Table 1, Fig. 1).

**Table 1:** Clinical and molecular data of individuals with bi-allelic variants in *CELSR3*

	Individual	ZYG	Variant	MAF	CADD; P; S; M	ID / DD	Tonus	Seizures	Brain, NTD	OFC	CAKUT	Other
**CNS**	**1: II-1**	CH	c.1574G>A, p.(Arg525His)	2.31e-5	27.5; 3; D; T	DD	np	np	np	-2.21 z	np	Atopic dermatitis, frequent febrile infections
			c.9299G>C, p.(Gly3100Ala)	NR	24.8; 2; D; HT							
	**2: II-1**	CH	c.7423C>T, p.(Arg2475Trp)	6.57e-5	28.1; 3; D; N	ID, DD	Central hypotonia	BNS seizures, DEE	np	np	np	Neonatal hypoglycaemia, TTTS (acceptor), FTT, facial dysmorphism
			c.8758C>T, p.(Arg2920Trp)	1.97e-5	25.7; 1; D; HT							
	**2: II-2**	CH	c.7423C>T, p.(Arg2475Trp)	6.57e-5	28.1; 3; D; N	ID, DD	Central hypotonia	BNS seizures, DEE	Delayed opercularisation	np	np	Neonatal hypoglycaemia, TTTS (donor), FTT, facial dysmorphism
			c.8758C>T, p.(Arg2920Trp)	1.97e-5	25.7; 1; D; HT							
	**3: II-1**	Hom	c.6304G>A, p.(Ala2102Thr)	NR	24.1; 1; D; SI	Global DD	Hypotonia	LGS	Prominence of subarachnoid spaces	np	X	Strabismus, sleep disturbance
	**4: II-3**	CH	c.5059C>T, p.(His1687Tyr)	1.97e-5	23.6; 1; D; SI	ID	Hypotonia	Seizures	Pachygyria, double cortex	np	X	X
			c.7075C>T, p.(Pro2359Ser)	1.98e-5	23.3; 2; T; SI							
	**5: II-2**	CH	c.7224_7226del, (p.Ile2409del)	1.32e-5	25.3; NR; NR; I	PSM-R	np	np	X	np	X	Autoaggressivity, autism-spectrum-disorder, stereotypies, pectus excavatum, facial dysmorphism
			c.8480C>A, (p.Thr2827Asn)	NR	17.8; 3; D; T							
	**6: II-1**	Hom	c.7999G>A, p.(Gly2667Ser)	NR	26.5; 3; D; I	ID, DD	np	np	Hairy naevus lumbal	np	np	Obesity, facial dysmorphism, stereotypies, sleep disturbance
**CNS**+**CAKUT**	**7: II-1**	Hom	c.6959T>C, p.(Val2320Ala)	NR	27.2; 3; D; SI	np	np	np	Polymicrogyria, subependymal heterotopia	np	MCDK, contralateral compensatory hypertrophy	X
	**8: II-1**	Hom	c.4034C>T, p.(Pro1345Leu)	2.63e-5	22.8; 1; T; SI	DD	Hypotonia and joint laxity	Generalized seizures	Obstructive hydrocephalus, ACC, cerebral hypoplasia	+2.31 z	Unilateral ectopic kidney	Rib deformities, ASD, sensorineural hearing loss, growth retardation, facial dysmorphism
	**9: II-1**	CH	c.3712C>T, p.(Arg1238Cys)	6.57e-6	28.2; 3; D; I	ID, DD	Asymmetric motor exam	Complex febrile seizures	Chiari I, L, periventricular cysts, fatty filum / TCS	+1.55 z	Duplicated collecting system, irregular bladder wall	Anxiety, facial dysmorphism
			c.7501G>A, p.(Glu2501Lys)	3.94e-5	25; 3; D; I							
**CAKUT**	**10: II-1**	Hom	c.3142C>T, p.(Arg1048Trp)	3.29e-5	23; 3; D; SI	np	np	np	X	np	Bilateral VUR, duplicated collecting system	X
	**11: II-1**	Hom	c.3100G>C, p.(Glu1034Gln)	7.23e-5	23.3; 2; T; I	np	np	np	X	np	Bilateral UPJO, hydronephrosis, reduced kidney function, diffuse bladder wall thickening	X

Overview of clinical and molecular data of twelve individuals from eleven independent families with bi-allelic variants in *CELSR3*. Further details, as well as information on three families which are not included in the main text, can be found in Supplement A and B.

*CNS* central nervous system, *CAKUT* congenital anomalies of the kidneys and urinary tract, *ZYG* zygosity, *CH* compound heterozygous, *Hom* homozygous, *MAF* minor allele frequency in gnomAD v3.1, *NR* not reported, *P* PolyPhen-2 (*1* benign, *2* possibly damaging, *3* probably damaging), *S* SIFT (*T* tolerated, *D* deleterious), *M* MetaDome (*HT* highly tolerant, *T* tolerant, *N* neutral, *SI* slightly intolerant, *I* intolerant), *ID* intellectual disability, *DD* developmental delay, *PSM-R* psychomotor regression, *BNS* Blitz-Nick-Salaam, *DEE* developmental and epileptic encephalopathy, *LGS* Lennox-Gastaut syndrome, *NTD* neural tube defect, *ACC* agenesis of corpus callosum, *L* leukoencephalopathy, *TCS* tethered cord syndrome, *OFC* occipitofrontal circumference, *MCDK* multicystic dysplastic kidney, *VUR* vesicoureteral reflux, *UPJO* ureteropelvic junction obstruction, *TTTS* twin-to-twin transfusion syndrome, *FTT* failure to thrive, *ASD* atrial septal defect, *np* phenotype not present, *X* not investigated.

Individuals with predominant CNS or combined CNS and CAKUT phenotypes presented with intellectual disability and/or developmental delay (ID / DD), hypotonia, seizures, brain malformations, NTDs, macro- or microcephaly (occipitofrontal circumference ±2 SD). The CAKUT spectrum in individuals with combined CNS and CAKUT or CAKUT only comprised duplicated collecting system, ectopic kidney, multi-cystic dysplastic kidney, vesico-ureteric reflux, hydronephrosis, obstructive uropathies or irregular bladder wall. Detailed phenotype information can be found in Table 1 and Supplement B.

### In silico analysis predicts intolerance of *CELSR3* variants

In individuals with a predominant CNS phenotype, eight out of ten variants were characterized as presumably damaging by at least two in silico prediction tools. One of these ten variants was predicted to be presumably damaging by all three prediction tools. All these variants affect residues highly conserved among species (Fig. 1a) and are annotated with a CADD score above 22, except for c.8480C>A (CADD score 17.8) (Table 1).

In individuals with CNS and CAKUT, and CAKUT only phenotype, five out of six identified variants were characterized as presumably damaging by at least two in silico prediction tools, four of these six variants were predicted to be presumably damaging by all three prediction tools. All these variants are annotated with a CADD score above 22 (Table 1) and affect highly conserved residues, except for c.3142C>T (Fig. 1b).

### Structural modeling reveals phenotype associated distribution of CELSR3 protein variants

**Fig. 2:**
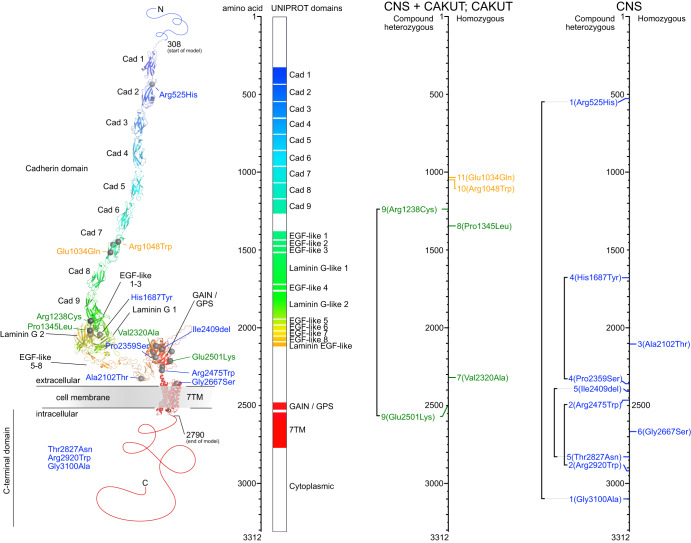
Structural modeling of CELSR3 and mapping of the variants. Structural modeling of CELSR3 and the respective variants according to the amino acid (aa) position. Left panel: 3D protein domain view and variant annotation using AlphaFold and PyMOL. Middle panel: Linearized aa view of the protein domains. Right panels: Variant location according to the respective phenotype categories: Central nervous system (CNS) anomalies in blue, combined CNS and congenital anomalies of the kidneys and urinary tract (CAKUT) in green, CAKUT only in yellow. Cad Cadherin, EGF Epidermal growth factor, GAIN G-protein-coupled receptor (GPCR) autoproteolysis-inducing domain, GPS GPCR proteolysis site, 7TM Seven-transmembrane.

We used PhosphositePlus^16^ and AlphaFold^17^ to create a model of the 3312 amino acid (aa) human CELSR3 protein using a ´divide-and-conquer´ strategy. The N-terminus of the protein comprises 307 aa (1–307) and the C-terminus contains 522 aa (2790–3312) for which no structural modeling was possible as no suitable homology template exists. CELSR3 contains seven membrane spanning helices (aa 2541–2774), which are part of the modeled section of this protein. The location of variants was mapped onto the model (Fig. 2). According to this modeling most modeled variants are predicted to potentially destabilize the respective region or affect the possible interaction surface due to changes in polarity or structure (Supplementary Table 1).

Only three of the ten variants found in individuals with a predominant CNS phenotype localize N-terminal in distance to the membrane associated domains. Whereas seven out of ten possibly CNS associated variants cluster within the perimembraneous domains and in the intracellular C-terminal domain (Fig. 2). Remarkably, the p.Ile2409del variant introduces a register shift into the side chain up-down sequence of a beta strand, potentially leading to a larger structural disturbance in this area. The p.Gly2667Ser variant is in an extracellular loop of the transmembrane domain and the variation to a polar serine can change the interaction surface of this region.

In comparison, all variants identified in individuals with CAKUT reside within extracellular N-terminal domains, including individuals 10: II-1 and 11: II-1 with variants in similar positions in one of the Cadherin domains (p.Arg1048Trp and p.Glu1034Gln) and similar CAKUT only phenotype (Table 1; Fig. 2). Two of these six possibly CAKUT associated variants cluster close to the GAIN-GPS motif: p.Val2320Ala, p.Glu2501Lys. The variant p.Val2320Ala might induce conformational changes of that loop in the GAIN domain and p.Glu2501Lys could significantly affect interactions by changes in polarity. Due to the absence of available research data, it was not possible to structurally model the cytoplasmic domains of CELSR3 (>500 aa). Interestingly, three of the in total 16 variants in twelve individuals identified in this study are located in this comparably small cytoplasmic area of the protein, suggesting this unresearched region to be important for protein function as well (Fig. 2).

### Detection of CELSR3 in the human embryonic metanephric kidney and urinary tract

**Fig. 3:**
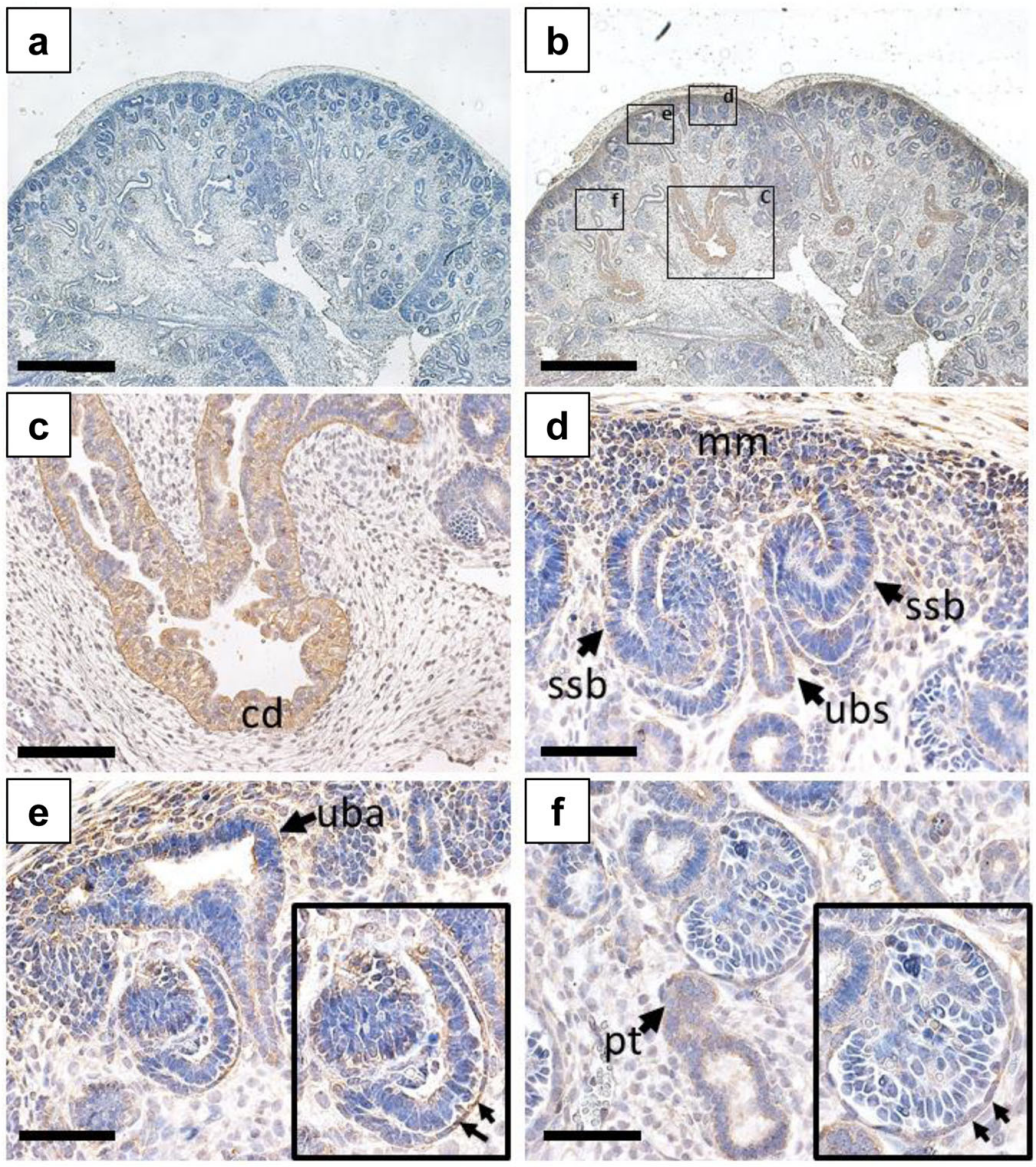
CELSR3 immunostaining in the human embryonic metanephric kidney at ten weeks gestation. All frames depict a ten-week gestation kidney with nuclei counterstained (blue) with hematoxylin. **a** Low power view of midsagittal section with primary antibody omitted. The nephrogenic cortex is uppermost and the medulla is in the low part of the image. Note the absence of brown color. **b** Adjacent section to that depicted in **a**. but immuno-stained for CELSR3. Note the positive signal *(brown)* in diverse structures. Boxed areas are detailed in **c**-**f**. **c** CELSR3 was detected in branching medullary collecting ducts *(cd)*. **d** The nephrogenic cortex contains immature structures. CELSR3 was detected in the ureteric bud branch stalk *(ubs)* which is flanked by nephron precursors called S-shape bodies *(ssb)*. The metanephric mesenchyme *(mm)* stained weakly for CELSR3. **e** Another view of the nephrogenic cortex showing the ureteric bud branch ampullary tip *(uba)*. These epithelia were weakly positive for CELSR3. Lower in the same image is an immature glomerulus with prominent CELSR3 immunostaining in the Bowman capsule, or parietal epithelia (arrows in the boxed enlargement). **f** The Bowman capsule of a more mature glomerulus has downregulated CELSR3 (arrows in boxed enlargement), and there is weak immunostaining in a nearby proximal tubule *(pt)*. Bars are 2 mm in frames **a** and **b**, and 200 µm in frames **c**–**f**.

CELSR3 was immuno-detected in different structures of the human embryonic metanephros, the precursor of the human kidney (Fig. 3, Supplementary Fig. 2). Similar patterns of CELSR3 were noted in metanephric kidneys at ten and twelve weeks of gestation. CELSR3 was detected in medullary collecting ducts and in ureteric bud branch stalks in the cortex of the developing organ. The protein was further detected in the Bowman capsule of immature glomeruli and there was weak immunostaining in proximal tubules. At the same stage, uncondensed metanephric mesenchyme in the outer cortex immuno-stained for CELSR3 as well (Fig. 3). In sections of a seven-week human embryo, CELSR3 was immuno-detected in epithelia of both the urogenital sinus tube, the precursor of the bladder urothelium, and also epithelia of the hindgut (Supplementary Fig. 2). The seven-week metanephric kidney contains a central ureteric stalk, with its branch tips capped by condensing metanephric mesenchyme, containing the nephron precursor cells. Neither of these showed a significant signal for CELSR3. But uncondensed metanephric mesenchyme in the seven-week metanephros stained for CELSR3. In addition, large (proximal) tubules in the adjacent mesonephros immuno-stained for CELSR3 as well (Supplementary Fig. 2).

### Transient suppression of CELSR3 ortholog Celsr3 in zebrafish leads to anomalies in the developing CNS and urinary system

**Fig. 4:**
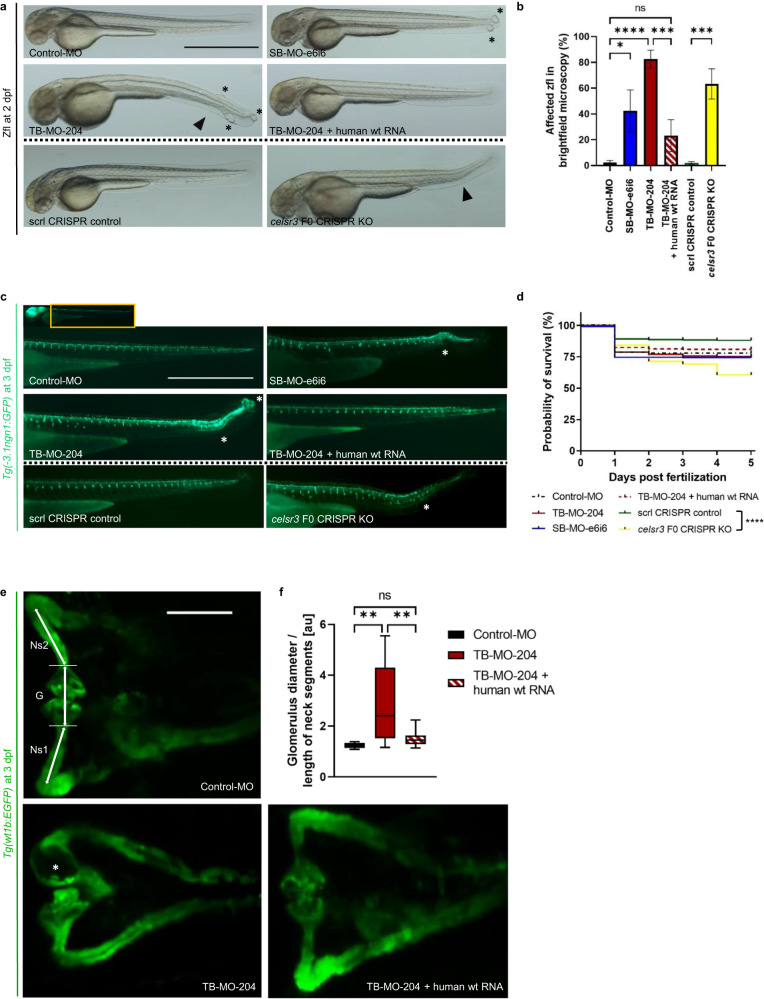
Transient suppression of Celsr3 in zebrafish larvae. Phenotypic evaluation of the different zebrafish larvae (zfl) groups: Zfl injected with Control-Morpholino (Control-MO), zfl injected with MO blocking *celsr3* splice site exon 6 – intron 6 (SB-MO-e6i6), zfl injected with MO blocking transcript *celsr3-204* (TB-MO-204), zfl co-injected with TB-MO-204 and human wild-type (wt) *CELSR3* polyA mRNA, zfl injected with scrambled (scrl) CRISPR control and *celsr3* F0 CRISPR knockout (KO) mixes. **a** Representative brightfield microscopy of laterally mounted zfl at two days post fertilization (dpf) treated with 1-phenyl 2-thiourea (PTU). Asterisks: Example caudal end disruption. Arrowhead: Example warped tail. Scale bar 1000 µm. **b** Percentage of affected zfl in brightfield microscopy. TB-MO-204 injected zfl and *celsr3* F0 CRISPR KO zfl show highly significant affection of a warped tail and/or caudal end disruption. In most zfl exposed to TB-MO-204 the phenotype could be rescued with human wt polyA *CELSR3* mRNA. Number (n) of zfl for each injection group: Control-MO (*n* = 157), SB-MO-e6i6 (*n* = 278), TB-MO-204 (*n* = 223), TB-MO-204 + human wt RNA (*n* = 221), scrl CRISPR control (*n* = 416) and *celsr3* F0 CRISPR KO (*n* = 440). Number of independent experiments *N* = 3 for both MO and CRISPR. **c** Representative laterally mounted *Tg(-3.1ngn1:GFP)* zfl at three dpf treated with PTU and imaged from lateral to visualize the effect of Celsr3 MO knockdown (MO-KD) or F0 *celsr3* KO on neurogenesis. The structural irregularities at the caudal end of the MO-KD or F0 *celsr3* KO zfl correlate with a disruption of the neuronal arrangement (white asterisks). Scale bar 1000 µm. **d** Kaplan–Meier plot showing a comparable survival rate for each respective injection group. Number (n) of zf embryos for each injection group: Control-MO (*n* = 203), SB-MO-e6i6 (*n* = 367), TB-MO-204 (*n* = 290), TB-MO-204 + human wt RNA (*n* = 272), scrl CRISPR control (*n* = 469) and *celsr3* F0 CRISPR KO (*n* = 611). Number of independent experiments *N* = 3 each. **e** Representative dorsally mounted *Tg(wt1b:EGFP)* zfl at three dpf treated with PTU and imaged from dorsal to visualize the effect of Celsr3 MO-KD on the development of the pronephros. White asterisk: Example enlarged glomerulus. G: Glomerulus. Ns1: Right neck segment. Ns2: Left neck segment. Scale bar 100 µm. **f** Box plot showing the size of the glomerulus in relation to the neck segments (G/((Ns1 + Ns2)/2)) calculated for each *Tg(wt1b:EGFP)* zfl at three dpf. MO-injected zfl show a highly significant increase of the glomerular diameter in comparison to the length of the neck segments. This effect was almost completely rescued when TB-MO-204 was co-injected together with human *CELSR3* wt polyA mRNA. Control-MO (*n* = 22), TB-MO-204 (*n* = 27), TB-MO-204 + human wt RNA (*n* = 29). Number of independent experiments *N* = 3. *p-value < 0.05, **p-value < 0.01, ***p-value < 0.001, ****p-value < 0.0001, *ns* not significant. Two-way ANOVA. Mean: SEM.

The similarity between the human CELSR3 protein and the zebrafish (zf) ortholog Celsr3 regarding all four described zf transcripts (*celsr3-201*, *celsr3-202*, *celsr3-203* and *celsr3-204*) is ~78% (2316/2959 aa) (SerialCloner 2.6.1 software). PCR amplification of the 5’ end of *celsr3* from zf cDNA revealed a larger transcribed region of at least an additional 2,138 base pairs (bp) compared to that previously described *celsr3-204*. Morpholino® knockdown (MO-KD) with the translational-blocking MO (TB-MO) targeted to the start codon 195 bp upstream of zf mRNA-transcript *celsr3-204* (TB-MO-204) as well as the splice-blocking MO (SB-MO-e6i6) both showed a matching phenotype during the first five days post fertilization (dpf) (Fig. 4, Supplementary Fig. 3). We defined the phenotype as a warped tail partly in combination with a disruption of neuronal or musculoskeletal tissue at the caudal end from two dpf onwards. This phenotype was significantly more frequent in SB-MO-e6i6-treated zf larvae (zfl) (42%) and TB-MO-204-treated zfl (83%) compared to Control-MO-treated zfl (2%, two-way ANOVA, *p* as indicated) (Fig. 4a, b, Supplementary Fig. 3). The co-injection of TB-MO-204 with human wild-type (wt) *CELSR3* polyA mRNA reduced this phenotype to 23% of zfl. Hence in most TB-MO-204-treated zfl the phenotype could be rescued with human wt mRNA of *CELSR3*. Furthermore, we could show that there is no significant difference between rescued and Control-MO-treated zfl.

In a parallel approach, we injected a CRISPR-Cas9 mix into zf embryos with six sgRNAs targeting *celsr3*. A comparable phenotype to Celsr3 MO-KD zfl could be replicated in these *celsr3* F0 CRISPR knockout (KO) zfl, as well as a significant increase of affected zfl (63%) compared to scrambled controls (2%) (Fig. 4a, b). Of note, no significant differences in survival rates among MO-KD, rescue and control groups were observed within the first five dpf. However, *celsr3* F0 CRISPR KO zfl presented with a lower survival rate compared to scrambled controls (Fig. 4d). Using transgenic *Tg(-3.1ngn1:GFP)* zfl at three dpf we visualized the disrupted arrangement of proliferating neuronal progenitor cells and decreased axonal outgrowth in Celsr3 KD or KO zfl (Fig. 4c). We further evaluated the structural development of the pronephros in transgenic *Tg*(*wt1b:EGFP)* zfl at three dpf (Fig. 4e, Supplementary Fig. 3). Here, TB-MO-treated zfl showed a significant dilatation of the glomerulus and a reduced size of the neck segments, compared to controls. This effect was almost completely rescued after co-injection of TB-MO-204 together with human wt *CELSR3* polyA mRNA (Fig. 4f).

## Discussion

In this study, we report twelve individuals from eleven independent families with rare or novel bi-allelic variants in *CELSR3* (Fig. 1, Table 1), most of them are missense variant alleles by conceptual translation.

Seven of ten variants of individuals with a predominant CNS phenotype reside in the intracellular or the peri-membranous protein region, including the GAIN and GPS domain (Fig. 2). The highly conserved GPCR-Autoproteolysis Inducing (GAIN) domain is structurally and functionally linked to the GPCR-Proteolysis Site (GPS) by mediating a chemical environment in the GPS necessary for autoproteolysis^18^. The intracellular C-terminal fragment (CTF) in GPCRs was found important for receptor density on the cell surface^19^, PCP^20^, and neural tube development^12,21,22^. Structural variation in the intracellular CTF of CELSR3 might impair receptor signaling predominantly leading to a CNS phenotype. Interestingly, all variants of individuals with a combined CNS and CAKUT or CAKUT only phenotype distribute within the extracellular domains. The extracellular cadherin repeats of CELSRs have adhesive properties and provide a likely structural mechanism for calcium-regulated interaction^4,5^. Our computed model of the human CELSR3 protein predicted potential structural and functional disturbances as a potential consequence of these respective extracellular or transmembrane variations (Fig. 2 and Supplementary Table 1). While these domains are structurally well characterized and allow quite precise calculation, intracellular variants are limited in structural interpretation due to sparse common information on the three-dimensional structure of the CTF. As indicated in the introduction, (rare) monoallelic variants in *CELSR3* have been described to be involved in neural tube defects (NTDs)^4,12^, febrile seizures^13^ and Tourette disorder^14^. However, the respective studies do not provide functional evidence to support this associations beyond doubt. Based on all available data, the protein was modeled and the effects of the identified variants were hypothetically outlined, implying certain limitations. Nevertheless, a limiting factor for the disease-gene relationship is that uncertainty remains about the specific function of the respective variants in the disease formation. Here identification of a larger cohort of affected biallelic variant carriers for further assessment is warranted. Furthermore, exploration of the respective variants in functional studies and cellular models are a direction for future research.

Previous studies indicated expression of Celsr3 protein in the CNS and disrupted axonal guidance in the forebrain of *Celsr3* conditional KO mice^8–10^. This led to a variety of developmental phenotypes of the CNS in these mice, which is in line with the phenotypic variability observed in our patients. While expression of Celsr3 in the developing CNS of mice was described previously^8–10^, we extended the expression profile of *CELSR3/Celsr3* to the embryonic and fetal bladder precursor tissues using human and mouse transcriptome data (GEO accession ID: GSE190641; Supplementary Fig. 1)^23^. These findings suggest a conserved role of *CELSR3/Celsr3* not only during CNS but also during urinary tract development in vertebrates (Supplementary Fig. 1). Furthermore, we immuno-detected CELSR3 in different embryonic structures of the developing human metanephric kidney and the urogenital sinus epithelium of the nascent bladder (Fig. 3 and Supplementary Fig. 2). The postulate that CELSR3 has critical roles in the growth and differentiation of these diverse cell types is consistent with the range of malformations described here (i.e., dysplastic and fused collecting systems).

In order to investigate not only expression but also the role of CELSR3 in development, we chose the zfl as a model organism. The similarity of the human CELSR3 protein compared to the zf ortholog Celsr3 is notably high with 78% (2316/2959 aa). The PCP core component pathway is mediated in part by the Celsr1-3 subfamily, conserved through mice and zf^24^. Relative expression data in zfl from one hour post fertilization (hpf) to 21 dpf indicated the highest expression of *celsr3* at three dpf^24^. Therefore, we examined the function of Celsr3 during early development in a MO-KD and F0 CRISPR-Cas9 KO zf model. The zf transcript *celsr3-204* is described to begin protein synthesis with glutamate (GAA), even though protein synthesis is initiated commonly with AUG methionine codons^25^. The AUG translational start site of transcript *celsr3-204* that we detected 195 bp upstream of the previously described beginning of exon 1 shows a high similarity to the human 5‘ translational start site of *CELSR3* and a strong Kozak consensus (Kozak: ACCAUGGCG; *celsr3-204* minus 195 bp: AGCAUGGAG). Therefore, we designed a TB-MO targeting this probable AUG translational start site of transcript *celsr3-204* (TB-MO-204).

Transient suppression of *celsr3* transcripts in fluorescent zfl reporter lines demonstrates the function of Celsr3 during early embryonic CNS and urinary tract development (Fig. 4, Supplementary Fig. 3). Previously, the expression of *celsr3* has been described in primary neural clusters of the brain and the spinal cord in zfl starting at twelve hpf^26^. We characterized the effect of Celsr3 MO-KD and *celsr3* F0 CRISPR KO on neurogenesis using the fluorescent reporter line *Tg(-3.1ngn1:GFP)* (Fig. 4c). The structural irregularities at the caudal end and disrupted neuronal migration in zfl morphants possibly resemble the neuronal anomalies described in individuals 2: II-2, 4: II-3, 6: II-1, 7: II-1, 8: II-1 and 9: II-1 (Table 1). Since five of the here described individuals presented with CAKUT, we chose the transgenic *Tg(wt1b*:*EGFP)* zf line as a vertebrate model system to analyze the effect of Celsr3 MO-KD on the early urinary tract development^27^. These zfl showed structural anomalies of the developing pronephros in Celsr3 zf morphants at three dpf (Fig. 4e, f, Supplementary Fig. 3). We classified the disproportionally enlarged glomerulus as a marker for disturbed development of the pronephros and the urinary tract, comparable to the kidney anomalies including hydronephrosis, obstructive uropathies and other CAKUT phenotypes observed in this study (Table 1).

In conclusion, the presented human genomic and immunohistochemical results, computational simulation of protein structure, and functional studies in zfl, collectively support the hypothesis that bi-allelic variants in *CELSR3* are involved in a probable genetic disease mainly affecting the CNS and urinary tract.

## Methods

### Ethics declaration

This study fulfilled the requirements of the Declaration of Helsinki and was approved by the Ethics Committee of the Medical Faculty of the University of Bonn (Lfd.Nr.031/19). Consent was obtained for all families including photographs if published, according to the respective research protocol of each institution. Human embryonic and fetal samples were surgically extracted from terminated pregnancies after informed consent and ethics approval. Mouse embryonic tissues have been documented and their usage reported to the local authorities (Regierungspräsidium Darmstadt). Human embryonic tissues were collected after maternal consent and with ethical approval of the North East - Newcastle & North Tyneside 1 Research Ethics Committee (REC18/NE/0290, https://www.hdbr.org). Animal husbandry and experimental setups were in accordance with European Legislation for the Protection of Animals used for Scientific Purposes (Directive 2010/62/EU). National law exempts all zebrafish experiments performed in larval stages up to five dpf before feeding from ethical approval.

Family 1: Written informed consent was obtained from the parents or legal guardians of the study participants after approval from the institutional review board (IRB) at the participating institutions. Approval EK302-16 was granted by the ethics committee of the Medical Faculty of the RWTH Aachen (Universitätsklinikum Aachen, Germany).

Family 2: All individuals or their families have signed written consent including clinical images, approved by the Ethics Committee of the Medical University of Innsbruck. Ethics application number UN4501.

Family 3: All individuals or their families have signed written consent. The study was approved by the IRB protocol 12-009346.

Family 4: All individuals or their families have signed written consent. The individual has been enrolled in a study for sequencing analysis after IRB approval at Policlinico S. Orsola-Malpighi (Bologna, Italy). IRB protocol 3206/2016.

Family 5: All individuals or their families have signed written consent through the Telethon Undiagnosed Disease Program (Naples, Italy).

Family 6: Written consent was obtained for publication of anonymized medical data which were obtained in a diagnostic setting. The affected individual was investigated by their referring physicians and all genetic analyses were performed in a diagnostic setting. Legal guardians of the affected individual gave informed consent for genomic investigations and publication of their anonymized data. For the Erasmus MC, use of genome-wide investigations in a diagnostic setting was IRB approved. IRB protocol METC-2012-387.

Family 7: All individuals or their families have signed written consent. This study was approved by Baylor College of Medicine (Houston, USA). IRB research Protocol H-29697.

Family 8: All individuals or their families have signed written consent through the Telethon Undiagnosed Disease Program (Naples, Italy). This study was approved under protocol number UDP15001. The authors affirm that human research participants provided informed consent for publication of the image in Fig. 1e.

Family 9: All individuals or their families have signed written consent. Agreement to perform Exome Sequencing was obtained by GeneDx (Gaithersburg, USA). Informed consent for publication was obtained from the family by the clinicians and standard permission form to photograph for academic and research purpose was signed.

Family 10: All individuals or their families have signed written consent. Approval for human subjects’ research was obtained from the IRB of the University of Michigan and Boston Children’s Hospital (Boston, USA). IRB protocol P00006200.

Family 11: All individuals or their families have signed written consent. Approval for human subjects’ research was obtained from the IRB of the University of Michigan and Boston Children’s Hospital (Boston, USA). IRB protocol P00006200.

Supplementary Family 1: The individual has given written permission for the publication of the data. In Denmark this is not considered a research project and participation is exempt from IRB and ethics approval.

Supplementary Family 2: All individuals or their families have signed written consent. This study was approved by the Columbia University IRB. IRB protocol AAAO6702.

Supplementary Family 3: All individuals or their families have signed written consent including diagnostic and publication.

### Variant identification and classification

We describe twelve individuals with bi-allelic variants in *CELSR3*. Furthermore, we describe three families with bi-allelic *CELSR3* variants of uncertain significance in the supplemental material. All individuals were ascertained through clinical exome sequencing (ES) and GeneMatcher^28^. Informed consent was obtained for all cases with additional permission to publish clinical images, if included. Race and ethnicity were self-reported or collected from databases. All sequencing methods, molecular findings and clinical descriptions are stated in Supplement A and B.

All variant alleles refer to the sequence of ENST00000164024.5 (Ensembl release 107, reference sequence NM_001407.3) and were validated with Mutalyzer 2.0.35^29,30^. Due to the size of the gene, we only considered variants not reported homozygous in gnomAD v3.1 and with a gnomAD v3.1 minor allele frequency (MAF) ≤ 0.0001 in order to avoid bi-allelic cases by chance. All variants, and segregation when parents available, were validated by Sanger sequencing. Additional, less likely variants of unknown significance reported in the here described individuals are mentioned in Supplement A.

For in silico analysis, we used Combined Annotation Dependent Depletion GRCh38-v1.6 (CADD), Polymorphism-Phenotyping v2 (PolyPhen-2) and Sorting Intolerant From Tolerant (SIFT)^31–33^. MetaDome Version 1.0.1 was applied to transcript ENST00000164024.4 to generate a tolerance landscape, analyzing the variants based on the single nucleotide missense and synonymous variants from gnomAD v3.1 in the protein-coding region^34^. Evolutionary conservation of bp positions was estimated using ConSurf^35,36^.

### Structural modeling of CELSR3 protein and mapping of the variants

PhosphositePlus^16^ and AlphaFold^17^ were used to model the structure of CELSR3. The source code of the AlphaFold (deepmind) algorithm was downloaded from https://github.com/deepmind/alphafold. Since the human CELSR3 comprising 3,312 aa residues is too large to readily create a full-length model, we split the protein into overlapping subdomains that were individually used as inputs for AlphaFold. The models of the individual domains were structurally aligned using PyMOL. The boundaries of the individual subdomains of the CELSR3 protein with overlapping segments for structural alignment were: 1–240, 121–360, 241–720, 601–840, 721–1320, 1201–1440, 1321–1980, 1861–2100, 1981–2400, 2281–2520, and 2401–3312, respectively.

### CELSR3 immunostaining of the human embryonic metanephric kidney and urinary tract

Human embryonic tissues, collected after maternal consent and with ethical approval (REC18/NE/0290), were sourced from the Medical Research Council and Wellcome Trust Human Developmental Biology Resource (https://www.hdbr.org). Samples comprised gestational week 7, 10, and 12. Tissues were paraffin embedded, sectioned as described^37^, and immunostained with the following primary antibody: Rabbit polyclonal raised to the N-terminal region of human CELSR3 (1:50 dilution; ab189012 from Abcam). The primary antibody was detected with a secondary antibody (1:200 dilution, Goat Anti-Rabbit ab6720 from Abcam) and signals generated with a DAB (SK-4100) peroxidase-based method^37^.

### Zebrafish husbandry and embryo maintenance

Zf were maintained according to recommendations by Westerfield^38^ and the German national law (animal welfare act and § 11). Zfl of wt AB/TL strain, transgenic *Tg(-3.1ngn1:GFP)* (ZFIN ID: ZDB-TGCONSTRCT-070117-124), and *Tg(wt1b:EGFP)* (ZFIN ID: ZDB-TGCONSTRCT-071127-1) were obtained by natural spawning and raised at 28 °C in Danieau solution on 14 h light and ten hours dark cycle. All experiments were done on zfl at one to five dpf before independent feeding^39^.

### Morpholino® knockdown and mRNA rescue microinjections

The zf wt ortholog *celsr3* (ENSDARG00000055825) is described with four transcripts (*celsr3-204*: ENSDART00000145095.3; *celsr3-201*: ENSDART00000078334.6; *celsr3-202*: ENSDART00000131888.2; *celsr3-203*: ENSDART00000137391.2; Ensembl release 107)^29^. The sequences of transcript *celsr3-202* and *celsr3-203* in zf are short and completely covered by transcript *celsr3-201*. Furthermore, transcript *celsr3-201* and *celsr3-204* overlap in parts and would be most similar to the only mentioned human transcript *CELSR3-201* as a combined transcript. Hence, analysis of the 5´UTR region with the purpose of identifying an AUG translational start site was performed by extraction of total RNA from 35 zfl with TRIzol™ reagent (Thermo Fisher Scientific, Catalog No. 15596026) and rt-PCR using ProtoScript® II First Strand cDNA Synthesis Kit (New England BioLabs GmbH, Catalog No. E6560). cDNA Amplification of 2138 bp upstream of exon 1 to exon 2 transition of transcript *celsr3-204* was performed using forward primer 5´-GAGCACGGCGGAAGGAGTCG-3´ and reverse primer 5´-CTCTGTAATGATGAGCACCCGCAGC-3´. Celsr3 protein sequence analysis of other species was facilitated using SerialCloner 2.6.1 software. Celsr3 KD was performed using specific Morpholino® Oligonucleotides (MO) synthetized by GeneTools, LLC. Two TB-MOs were designed targeting the AUG translational start site of transcript *celsr3-201* (TB-MO-201, 5´-CTGCTGAGCATCTCCTCTGTAATGA-3´) and the expected AUG translational start site of transcript *celsr3-204* (*celsr3-204* minus 195 bp, TB-MO-204, 5´-GTCTTCTGCAATCACCCACTCCATG-3´). One splice-blocking MO was designed targeting the boundary of *celsr3-204* exon 6 – intron 6 (SB-MO-e6i6, 5´-TCTTCAGTGGACTTTCTCACCTTGT-3´). In one- to two-cell zf embryos, MO microinjections were performed into the yolk with *celsr3* TB-MO-201, *celsr3* TB-MO-204 or the standard control MO (Control-MO, 5´-CCTCTTACCTCAGTTACAATTTATA-3´) with ~4.5 ng for each MO (1.8 nl/embryo), or with ~5.9 ng for *celsr3* SB-MO-e6i6 (1.8 nl/embryo). For mRNA rescue experiments, ~70 pg of in vitro transcribed human wt *CELSR3* polyA mRNA and TB-MO-204 with non-identical sequences (bp) were co-injected. mRNA transcription was performed on human *CELSR3* cDNA ORF clone OHu18524 (GenScript) containing NM_001407.3 using the mMESSAGE mMACHINE T7 Ultra Kit (Thermo Fisher Scientific, Catalog No. AMB13455) followed by polyA tailing using Invitrogen™ Poly-(A) Tailing Kit (ThermoFisher, Invitrogen™, Catalog No. AM1350). For zfl injection, concentrations were chosen to avoid dose-dependent effects after examination ranges of ~3.7–7.4 ng MO and 20–100 pg/nl mRNA solution.

### CRISPR–Cas9 F0 knockout of *celsr3*

F0 KO zfl were generated using the CRISPR-Cas9 method as previously described with slight modifications to the protocol^40^. With the purpose of creating a truncated *celsr3* transcript we designed six sgRNAs binding shortly upstream of exon 1, in exon 1 and in exon 2 of *celsr3-204* (NM_001407.3) using the open website tool https://www.crisprscan.org/^41^. Reagents of Alt-R^TM^ CRISPR-Cas9 System (Alt-R® CRISPR-Cas9 crRNA, custom design; Alt-R® CRISPR-Cas9 tracrRNA; Alt-R® S.p. Cas9 Nuclease V3, Catalog No. 1081058; Alt-R® CRISPR-Cas9 Negative “scrambled” control crRNA #1-#3, Catalog No. 1072544-1072546; Nuclease-Free Duplex Buffer, Catalog No. 11-05-01-12) were obtained from Integrated DNA Technologies, Inc and prepared according to the distributor’s and user protocols^42^. Equal amounts of either all six *celsr3* sgRNAs or the three scrambled control sgRNAs were combined to a 100 μM stock. Equal amounts of these 100 µM sgRNA stocks and 100 µM tracrRNA were diluted in Nuclease-Free Duplex Buffer to a final concentration of 3 µM or 6 µM for scrambled control and *celsr3* mix and annealed at 95 °C for 5 min. These were combined with the same amount (3.05/6.1 µM) of Cas9 protein diluted in Cas9 working buffer each and incubated at 37 °C for 10 min. Shortly before injections 1 µl of phenol red was added. Injections of ~1.8 nl were performed into the yolk of one- to two-cell zf embryos. Truncation of the *celsr3* genomic region was PCR tested at four dpf as previously described^43^. Primer sequences, sgRNA sequences and genomic PCR-gel electrophoresis images are provided in Supplementary Fig. 4.

### In vivo imaging and phenotyping

Zf embryos were incubated with 0.2 mM 1-phenyl 2-thiourea (PTU, Catalog No. P7629) supplemented to their Danieau solution from one to five dpf to avoid pigmentation. Brightfield and fluorescence in vivo imaging was performed from one to five dpf using a ZEISS Axio V16 Multi-Zoom microscope and analyzed with ZEN 2.3 Software. Phenotypic affection in brightfield imaging was defined by the presence of irregular tail curvature and/or disruption at the caudal end at two dpf. For CNS phenotype evaluation *Tg(-3.1ngn1:GFP)* zfl at three dpf were anesthetized with 0.03% tricaine and fixed in 1.25% low-melting agarose. For evaluation of the urinary tract phenotype *Tg(wt1b:EGFP)* zfl at three dpf were anesthetized and fixed as described above. Z-stack-series with 2 µm step size was performed with a Nikon A1R HD25 ECLIPSE Ti2E confocal laser scanning microscope equipped with NIS-Elements 5.21.02 software. Phenotypic differences in the developing pronephros in *Tg(wt1b:EGFP)* were analyzed with the NIS-Element imaging software 5.21.00. To account for variation in larvae size, the glomerulus diameter was normalized to the respective length of the pronephric neck segments. All experiments were repeated independently at least three times (*N* ≥ 3).

### Statistical analyses

GraphPad Prism Version 9.0.0 was used for one-way ANOVA with post-hoc Tukey HSD Test and two-way ANOVA with SEM. Kaplan–Meier survival curves were used to analyze survival within the first five dpf.

### Reporting summary

Further information on research design is available in the Nature Research Reporting Summary linked to this article.

### Supplementary information

Supplemental material

REPORTING SUMMARY

## Data Availability

Data supporting the findings of this study are available in the supplemental material. Additional data not compromised by ethical issues will be available upon request from the corresponding authors. All sequencing data are deposited in ClinVar (accession numbers: SCV004176841 – SCV004176856). Mouse RNA-seq data at stages E10.5, E12.5 and E15.5 were obtained from Gene Expression Omnibus (GEO accession ID: GSE190641)^23^. RNA-seq data of human embryonic and fetal bladder tissues were obtained from already deposited data at EMBL-EBI expression atlas (E-MTAB-6592).
