# Supplementary material for: Bi-allelic variants in CELSR3 are implicated in central nervous system and urinary tract anomalies
Source: NPJ Genom Med. 2024 Mar 1;9:18. doi: 10.1038/s41525-024-00398-9 (PMC10907620; doi:10.1038/s41525-024-00398-9)
Supplement: Supplementary file 1 — Supplemental material [file 41525_2024_398_MOESM1_ESM.pdf]

SUPPLEMENTAL MATERIAL

Supplement A: Molecular studies, sequencing methods and data analyses

Family 1: Individual 1: II-1 is followed by the Medical Faculty of the RWTH Aachen University in Germany. ES revealed compound heterozygous variants in *CELSR3*. Both variants are autosomal recessive inherited, with c.9299G>C, p.(Gly3100Ala) inherited by the mother and c.1574G>A, p.(Arg525His) paternally inherited. All variants including the parents were confirmed by Sanger Sequencing.

Family 2: Individual 2: II-1 and individual 2: II-2 are twin sisters. Duo ES including both affected individuals and the healthy mother revealed compound heterozygous variants in *CELSR3*. Both variants were confirmed to be in trans, with c.7423C>T, p.(Arg2475Trp) inherited by the mother and c.8758C>T, p.(Arg2920Trp) inherited by the father. The variant and inheritance were confirmed by Sanger Sequencing.

Family 3: Individual 3: II-1 is followed by the Center for Individualized Medicine at the Mayo Clinic in Rochester, MN, USA. Manual re-analysis of clinical trio ES revealed a homozygous variant c.6304G>A, p.(Ala2102Thr) in *CELSR3*, autosomal recessive inherited by the parents. The variant and inheritance were confirmed by Sanger Sequencing. Prior testing including CMA showed extensive homozygosity but no CNVs. Expanded epilepsy panel was negative. Another homozygous variant in *LMX1A* (c.883A>T, p.T295S) was detected by the clinical lab and validated. This is a moderate substitution in a poorly conserved codon and is considered non-contributory to the individual’s condition, as *LMX1A* is not known to be involved in neurodevelopmental disorders besides deafness.

Family 4: Individual 4: II-3 is carrying compound heterozygous variants c.7075C>T, p.(Pro2359Ser) inherited by the mother and c.5059C>T, p.(His1687Tyr) inherited by the father in *CELSR3*, revealed with trio whole exome sequencing with Twist Human Core Exome Kit v.1.3. The variant and inheritance were confirmed by Sanger Sequencing as well as the heterozygous status of the two older unaffected sisters for c.7075C>T,

p.(Pro2359Ser). No other potential causative variants have been identified, especially no variants in genes known to cause double cortex or pachygyria.

Family 5: Individual 5: II-2 is followed by the Telethon Undiagnosed Diseases Program. Trio ES revealed compound heterozygous variants in *CELSR3*. The in-frame deletion c.7224_7226del, p.(Ile2409del) is maternally inherited and the missense variant c.8480C>A, p.(Thr2827Asn) is paternally inherited. The variants and inheritance were confirmed by Sanger Sequencing.

Family 6: Individual 6: II-1 is carrying a homozygous variant c.7999G>A, p.(Gly2667Ser) in *CELSR3*, and is followed at the Erasmus MC University Medical Center in the Netherlands. Trio ES was performed using Agilent SureSelect Clinical Research Exome (CRE) kit (v2) with a coverage of 320x of that region. Segregation analysis confirmed the *CELSR3* variant in individual 6: II-1 and showed that the *CELSR3* variant was absent in the unaffected brother. Additionally, there was normal Fragile X testing for 6: II-1 and large runs of homozygosity (ROH) regions given consanguinity of parents. Sequencing revealed three additional variants of unknown significance in genes without an OMIM phenotype: NM_001329630.2(PLEKHA7):c.1523C>T, p.(Ser508Leu). This variant was also homozygous in the apparently healthy brother. In GeneMatcher there was one other bi-allelic case with a multiple congenital anomaly phenotype not matching individual 6: II-1. NM_000482.3(APOA4):c.400C>T, p.(Arg134Cys). This variant was also homozygous in the apparently healthy brother. Besides, there was no other case in GeneMatcher. NM_003225.3(TFF1):c.71C>A, p.(Ala24Asp). This variant was heterozygous in the apparently healthy brother. Investigation in knockout mice did not show any organ abnormalities besides abnormalities of the stomach (Lefebvre et al. 1996: Gastric mucosa abnormalities and tumorigenesis in mice lacking the pS2 trefoil protein. In: *Science*). Besides, Individual 6: II-1 was also homozygous for NM_152783.4(D2HGDH):c.1475C>T, p.(Pro492Leu), but metabolic investigations were all normal and did not show evidence of D-2-hydroxyglutaric aciduria.

Family 7: Individual 7: II-1 is from a cohort of individuals with unsolved neurodevelopmental disorders studied in the Baylor Hopkins Centers for Mendelian Genomics rare disease research program. Proband-only ES was performed using the Baylor College of Medicine Human Genome Sequencing Center core capture (52 Mb) reagent using and sequenced on the Illumina HiSeq platform, achieving an average depth of coverage of 103X. ES analysis revealed a homozygous (39 variant reads out of 39 total reads) variant c.6959T>C, p.(Val2320Ala) in *CELSR3*. The variant was validated homozygous for 7: II-1 and heterozygous for the mother by Sanger Sequencing. No further likely disease-causing variants were identified.

Family 8: Individual 8: II-1 is followed by the Telethon Undiagnosed Diseases Program in Italy. Trio ES revealed a homozygous variant c.4034C>T, p.(Pro1345Leu) in *CELSR3.* Sanger Sequencing was validated for both, 8: II-1 and the heterozygous status of the parents. No further likely disease-causing variants were identified.

Family 9: Individual 9: II-1 is followed by the Riley Hospital for Children in Indiana. ES revealed compound heterozygous variants in *CELSR3* with c.7501G>A, p.(Glu2501Lys) inherited by the mother and c.3712C>T, p.(Arg1238Cys) inherited by the father. Sanger validation through GeneDx validated the variants and approved the inheritance of one copy from each parent. Additionally, a *de novo* deletion c.2240_2242del, p.(Val747del) in *EIF3B* was detected.

Family 10: Individual 10: II-1 is from a cohort of 822 individuals with CAKUT from 731 unrelated families. ES revealed a homozygous variant c.3142C>T, p.(Arg1048Trp) in *CELSR3*. No parental DNA was available for testing. The variant was confirmed by Sanger Sequencing. No further likely disease-causing variants were identified.

Family 11: Individual 11: II-1 is from a cohort of 822 individuals with CAKUT from 731 unrelated families. ES revealed a homozygous variant c.3100G>C, p.(Glu1034Gln) in *CELSR3*. No parental DNA was available for testing. The variant was confirmed by Sanger Sequencing. No further likely disease-causing variants were identified.

The following three individuals can only be reported with some uncertainty due to either incomplete phenotyping (Supplementary Family 1) or incomplete segregation and variant validation (Supplementary Family 2 - 3).

Supplementary Family 1: Supplementary Individual 1: II-1 is followed by the Aarhus University Hospital in Denmark. Trio ES revealed a homozygous variant c.1567G>A, p.(Gly523Arg) in *CELSR3*. The parents and the younger sister are heterozygous for the respective variant. The variant and inheritance were confirmed by Sanger Sequencing.

Supplementary Family 2: Supplementary Individual 2: II-1 is carrying two heterozygous *CELSR3* variants c.7493G>A, p.(Arg2498Gln) inherited by the mother and c.7886G>A, p.(Arg2629His) absent in the mother, revealed with clinical ES at GeneDx. There was no paternal DNA available for segregation. Therefore, it could not be clearly demonstrated that the two variants were compound heterozygous. Besides, there was no Sanger validation performed. Additionally, sequencing revealed one maternally inherited heterozygous variant in *MAP4K4* c.2619+2T>C and one heterozygous variant in *JMJD1C* c.3265C>T.

Supplementary Family 3: Supplementary Individual 3: II-1 is carrying two heterozygous variants variants in *CELSR3*, revealed with duo ES including maternal DNA at GeneDx. One variant c.8837A>G, p.(Asn2946Ser) is maternally inherited and was confirmed by Sanger Sequencing. The occurrence of the second variant c.9170G>A, p.(Gly3057Asp) is unknown. There is no paternal DNA available for segregation. Therefore, it could not be clearly demonstrated that the two variants were compound heterozygous. Additionally, there was a variant in *VWF* c.2561G>A, p.(Arg854Gln). Copy number loss at 13q12.12 was present in the mother.

Supplement B: Phenotype description of individuals

Family 1: Individual 1: II-1 is a four-year-old male of German descent. He has one healthy brother. 1: II-1 showed developmental delay with microcephaly. His head circumference was 48.5 cm (-2.21 z) at four years of age. His brain MRI was without pathological findings. His ultrasound examinations were clinically unremarkable regarding renal malformations, anorectal and dysraphic disorders. He did not present seizures, growth retardation or concise dysmorphic features. In addition, he was diagnosed with atopic dermatitis and frequent febrile infections in early childhood.

Family 2: Individual 2: II-1 and 2: II-2 are three-year-old female twins of Serbian descent. During pregnancy, twin-to-twin transfusion syndrome (TTTS) was diagnosed with 2: II-1 as the acceptor. The twins were born in the 35th week of gestation. Weight at birth was at 15th percentile, height at 19th percentile, and head circumference at 12th percentile in individual 2: II-1, and at 3rd percentile, 19th and 19th percentile, respectively, in the twin sister 2: II-2. Individual 2: II-1 presented with neonatal hypoglycaemia and central hypotonia. A diagnosis of developmental and epileptic encephalopathy (DEE) with hypsarrhythmia and seizure-associated athetosis and dystonia from birth, and subsequently developed Blitz-Nick-Salaam seizures was made. She showed global developmental delay with intellectual disability and developmental regression starting at the age of seven months. There was no evidence for structural brain malformations in her brain MRI at the age of eight months. Under ACTH treatment and ketogenic diet, she showed a seizure frequency of six events per day. Furthermore, she showed facial dysmorphism with frontal bossing and a large anterior fontanelle, thin lips, high arched palate, tent-like mouth and anteverted nostrils. Besides, she showed low visual fixation, and feeding difficulties. Her twin sister, individual 2: II-2 presented with a nearly identical picture and disease course. In addition, she showed delayed temporal opercularisation of the temporal lobe at the age of eight months. Extensive metabolic workup and a chromosomal microarray was unremarkable in both twins. Individual 2: II-1 and 2: II-2

had a normal renal ultrasound at birth and at eight months of age and normal renal function at one year of age.

Family 3: Individual 3: II-1 is a three-year-old male of Pakistani descent. He was born full term after a pregnancy complicated by hyperemesis gravidarum to unaffected, consanguineous parents (cousins of first degree). His brain MRI was not informative besides of a moderate generalized prominence of the subarachnoid spaces. He developed seizures of the Lennox-Gastaut syndrome (LGS) type in infancy. Additionally, he showed generalized hypotonia and global developmental delay including delayed speech. He had poor oral motor control, poor weight gain, strabismus, and sleep disturbance including snoring.

Family 4: Individual 4: II-3 is a 20-year-old female of Italian descent. She has two older unaffected sisters. Brain MRI revealed neuronal migration disorders including pachygyria and double cortex. At the age of 20 months she developed seizures with heterogeneous seizure semiology. Besides, she presented with hypotonia and moderate intellectual disability.

Family 5: Individual 5: II-2 is an 18-year-old male of Italian descent. He and his healthy older brother are born to presumed unrelated and healthy parents. He showed a normal development until the age of 18 months, then psychomotor regression started from speech and gradually involved all fields. He was able to hold his head at three months, to sit at six months, and to walk with support at 16 months. The first words were pronounced at twelve months, but afterwards speech development was very poor. At the age of 18 months, he experienced a psychomotor regression especially affecting language skills, leading to a loss of speaking ability. At the same age, he started to develop behavioral issues with limited interaction and autism-spectrum disorder features. At the age of 16 years, he could walk with support but was nonverbal and showed a very limited social interaction. He also presented with motor stereotypies, variable tics, and some frustration intolerance with auto aggressive behaviors. In addition, he showed tics and hand movements stereotypies when he was in agitation. Clinical evaluation revealed deep-set eyes, thick eyebrows, down-slanting palpebral fissures, protruding ear, underdeveloped superior crus of antihelix, short philtrum,

prominent nasal bridge, dental malocclusion, exaggerated cupid´s bow, thick vermilion border and pectus excavatum.

Family 6: Individual 6: II-1 is a five-year-old male of Afghan descent. He and his unaffected younger brother are born to consanguineous parents. He presented with developmental delay without speech development at the age of five years and poor interactions. He started independent walking at 13 months with a clumsy gait. Besides, he showed stereotypies and temper tantrums. He has a congenital hairy melanocytic nevus with a diameter of 10 to 15 centimeter at the level of the lower lumbal spine. Radiologic imaging of the brain or the spine was not performed. Abdominal ultrasound including the kidneys and urinary tract was reported to be normal. Clinical evaluation revealed a slightly coarse looking face and fleshy earlobes. In addition, obesity (+ 3.58 SDS) with normal metabolic screening, sleep disturbance, possible apnea and picky eating were reported.

Family 7: Individual 7: II-1 is an 18-year-old female of Mexican descent who presented with unilateral multicystic dysplastic kidney (MCDK) and contralateral compensatory hypertrophy. Brain MRI revealed cortical thickening and extensive frontal and parietal polymicrogyria. In addition, bilateral subependymal heterotopia was diagnosed. However, neurological symptoms were not reported to the point of last examination.

Family 8: Individual 8: II-1 is a ten-year-old female of Egyptian descent. She has one brother and one sister, both are unaffected. Furthermore, she has a cousin of first degree who showed global developmental delay, but he was not available for testing. The individual presented with a unilateral ectopic kidney in paravesical position, regular parenchyma and slightly reduced kidney volume. Brain MRI at the age of one month revealed aqueduct stenosis with hydrocephalus and was treated with a ventriculoperitoneal shunt. Additionally, an agenesis of the corpus callosum, hypoplasia of the cerebral hemispheres and suspected cerebellar malformations were detected. Clinical examination showed initially macrocephaly with cranial circumference of 56 cm (+ 2.31 z), high and prominent forehead and very small and low-set ears. She showed generalized tonic-clonic seizures and was treated with

Levetiracetam. In addition, she was diagnosed with hypotonia and joint laxity. The individual was diagnosed with developmental delay. She was only able to walk with support, was not able to speak, showed poor interaction to others and poor swallowing condition. The individual showed growth retardation with height and weight below 0.2nd percentile (< 3 SD). Besides, she showed scoliosis, rib deformities of the third and fourth left rib and the fourth right rib, ostium secundum atrial septal defect and pantonal sensorineural hearing loss.

Family 9: Individual 9: II-1 is a twelve-year-old male of Scottish descent. He was born at 36 weeks gestational age to presumed unrelated and healthy parents in a triplet gestation. There was intrauterine demise of one triplet. For his sister from the same pregnancy there is no phenotype or genotype reported. Individual 9: II-1 presented with a duplicated right renal collecting system. Besides, he showed an irregular urinary bladder wall, thought to be either neurogenic or under-distended. Head and neck MRI at the age of three revealed Chiari I malformations, status post decompression, and a patchy leukoencephalopathy with periventricular cysts. Spine MRI at the age of eleven revealed a fatty filum and a tethered cord, status post neurosurgical release. Besides, he presented with complex febrile seizures. At the age of 14, he had a head circumference of 57 cm (+1.55 z). His maximum deviation was at age three, when his head circumference was 52.5 cm (+1.85 z). He showed asymmetric motor exam, developmental delay, and an intellectual presentation little behind academically. His sister showed intellectual property above average for her grade. Further clinical examination of 9: II-1 showed minor dysmorphic facial features and a normal stature (z-score - 0.27 to - 1.02 since infancy). Additionally, he presented with anxiety.

Family 10: Individual 10: II-1 is a four-year-old female of Yemeni descent and was born to presumed unrelated and healthy parents. The individual’s sister is not affected. Clinical diagnostics revealed bilateral vesicoureteral reflux (VUR) grade four for the left and grade three for the right kidney and left sided duplicated collecting system. The individual is not reported to have any CNS malformation. Seizures were not reported. To the point of last examination, the individual showed age-appropriate growth and development. As this patient

was four years old at time of investigation, a detailed evaluation of development and milestone achievement was conducted clinically. As there were no abnormal observations, additional cranial imaging was clinically not indicated.

Family 11: Individual 11: II-1 is a seven-year-old female of Pakistani descent and was born to presumed unrelated and healthy parents. The individual was diagnosed with bilateral ureteropelvic junction obstruction (UPJO) with bilateral hydronephrosis and reduced kidney function. Ultrasound revealed a diffuse bladder wall thickening. There was no indication for any CNS malformation. Seizures were not reported. To the point of last examination, the individual showed age-appropriate growth and development. As this patient was seven years old at time of investigation, a detailed evaluation of development and milestone achievement was conducted clinically. As there were no abnormal observations, additional cranial imaging was clinically not indicated.

The following three individuals can only be reported with some uncertainty due to either incomplete phenotyping (Supplementary Family 1) or incomplete segregation and variant validation (Supplementary Family 2 - 3).

Supplementary Family 1: Supplementary Individual 1: II-1 is a deceased female newborn of Afghan descent. During pregnancy, polyhydramnios was detected and fetal movements were reduced in the last weeks of pregnancy. She was delivered in gestational week 38 with a weight of 2.1 kg (< 3rd percentile). She could not be ventilated due to solid secrets blocking the trachea, potentially caused by hypotonia. Besides, the complete forensic examination was unremarkable. Due to the unclear situation during birth, the phenotype cannot be sufficiently represented and there was no clear evidence of functional or structural abnormalities. In the following pregnancy of the same parents, a healthy daughter was born.

Supplementary Family 2: Supplementary Individual 2: II-1 is a four-year-old female of European descent. She presented with epilepsy characterized by generalized convulsions.

Clinical evaluation revealed hypotonia and motor delay including difficulties with chewing and swallowing. Besides, she presented with hypothyroidism.

Supplementary Family 3: Supplementary Individual 3: II-1 is an eleven-year-old female of European descent. She was born to presumed unrelated and healthy parents and has one maternal half-sister and paternal half-siblings. Renal ultrasound at the age of nine years was normal, but she showed a low volume urination and a history of recurrent urinary tract infections. Brain MRI was normal. She has a history of fixed eye gaze followed by lethargy suspicious for possible seizures. EEG recording without clinical events was unremarkable. She has had a one-time episode, the history of which is consistent with a generalized seizure, in the setting of an asthma exacerbation. Chest CT showed multiple scattered tiny pulmonary nodules bilaterally (seven total, measuring 1 – 4 mm each). Bronchoscopy showed adenoid hypertrophy, pharyngomalacia, glossoptosis, bronchitis, and fixed partial obstruction of the inferior aspect of the left mainstem bronchus. Besides, she presented with muscle cramps and fatigue. She showed global developmental delay in terms of not meeting major milestones, poor coordination, and an excoriation disorder. Intellectual scores were within the average range or higher, except for tests that emphasize visual-motor coordination and fine motor skills (which were below average). She was diagnosed with autism spectrum disorder. Clinical evaluation revealed down-slanting palpebral fissures and ptosis, normal eyebrows, bulbous nasal tip, depressed nasal bridge, and thin lips. Her X-ray showed advanced bone age and leg length discrepancy of 5 mm. Additionally, she presented with obesity, prediabetes, tall stature, anxiety, obstructive sleep apnea, precocious puberty, headaches, hyperphagia, joint pain, significant bruising with minimal trauma, flat feet, vocal cord dysfunction, and eczema.

Supplementary Figure 1

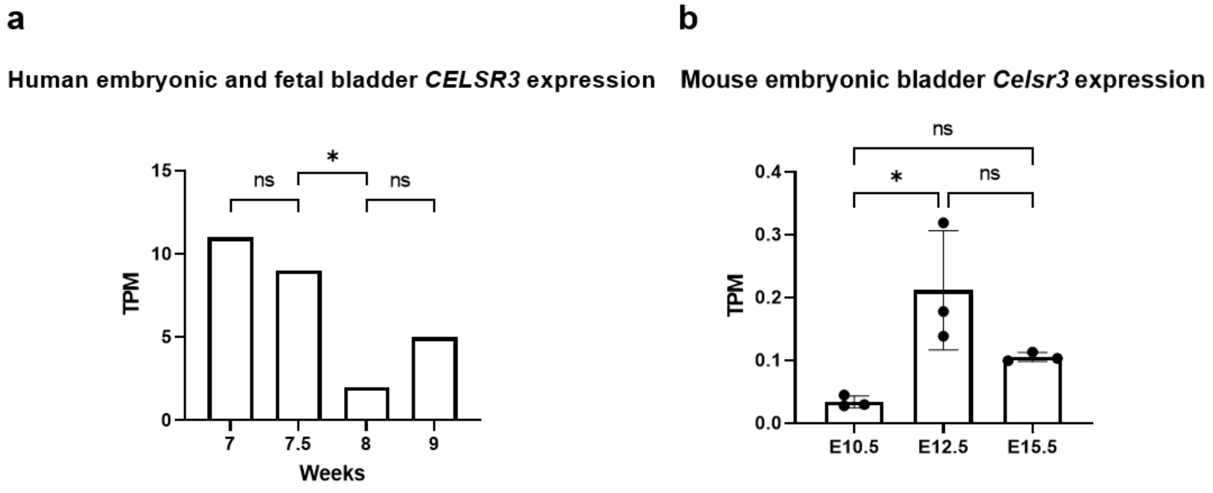

Human and Mouse transcriptome data in developing bladder tissues

Transcripts per million (TPM) expression level of *CELSR3* mRNA in developing bladder of human and mouse. *CELSR3* is expressed in both human and mouse bladder tissues. **a.** *CELSR3* expression in human embryonic and fetal bladder. Human samples comprising gestational weeks 7 (n = 2), 7.5 (n = 1), 8 (n = 3), and 9 (n = 4). *CELSR3* expression is significantly downregulated between week 7.5 and 8. **b.** *Celsr3* expression in developing mouse bladder. Transcripts are significantly upregulated from cloaca tissue (E10.5) to primitive bladder (E12.5). Fold-change (FC) was calculated dividing the subsequent stage for the preceding one and the $\log_2$ function was applied to the division as following: $\log_2 FC = \log_2$ (subsequent embryonic stage / preceding embryonic stage). Differential expression of genes was classified by FC ≤ -1.5 or ≥ 1.5. * p-value < 0.05. Two-way ANOVA. Mean with SD.

Supplementary Figure 2

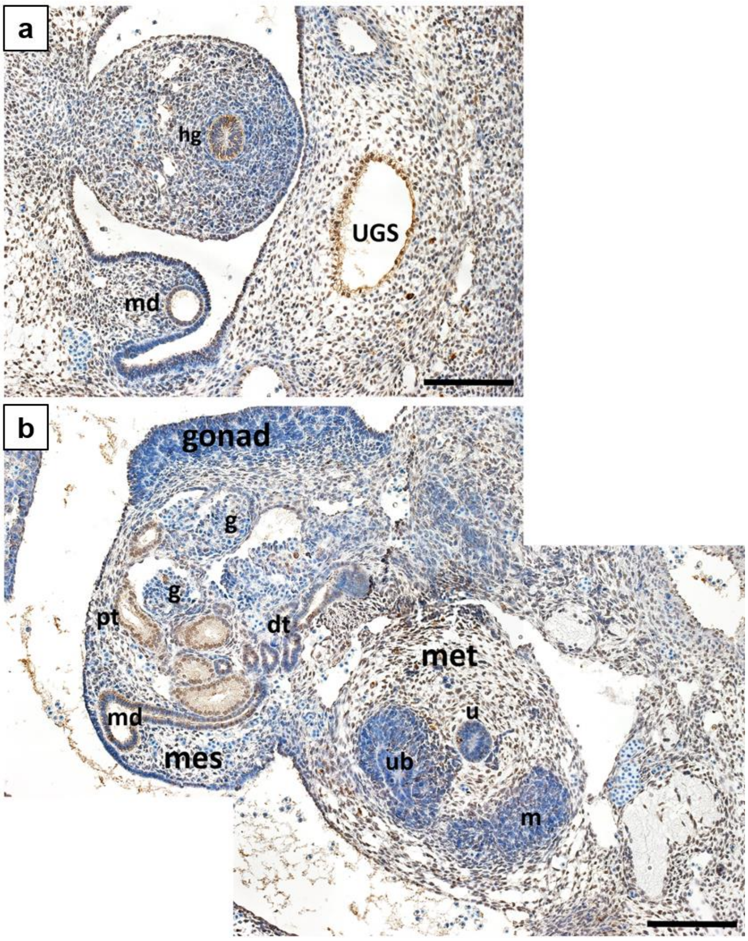

CELSR3 immunostaining in the human embryonic urinary tract at seven weeks gestation

Immunostaining of human seven-week embryonic sections for CELSR3 (brown) and nuclei counterstained (blue) with hematoxylin. **a.** Transverse section at the level of the urogenital sinus *(ugs)*. Note positive (brown) CELSR3 signal in the simple epithelium lining the *ugs*, and in the hindgut epithelium *(hg)*. The mesonephric duct *(md)* is indicated. **b.** This section shows the mesonephric kidney *(mes)* with its proximal tubules *(pt)* immunostained for CELSR3. Also indicated are mesonephric glomeruli *(g)*, distal tubules *(dt)* and mesonephric duct *(md)*. The gonadal ridge *(gonad)* is nearby. The adjacent metanephric kidney *(met)* is less differentiated than the mesonephros. Within the metanephros, uncondensed metanephric mesenchyme immunostained for CELSR3. Other structures are indicated: The ureteric stalk *(u)*, a ureteric bud branch tip *(ub)*, and mesenchyme *(m)* condensing around the branch tips. Bars are 100 µm.

Supplementary Figure 3

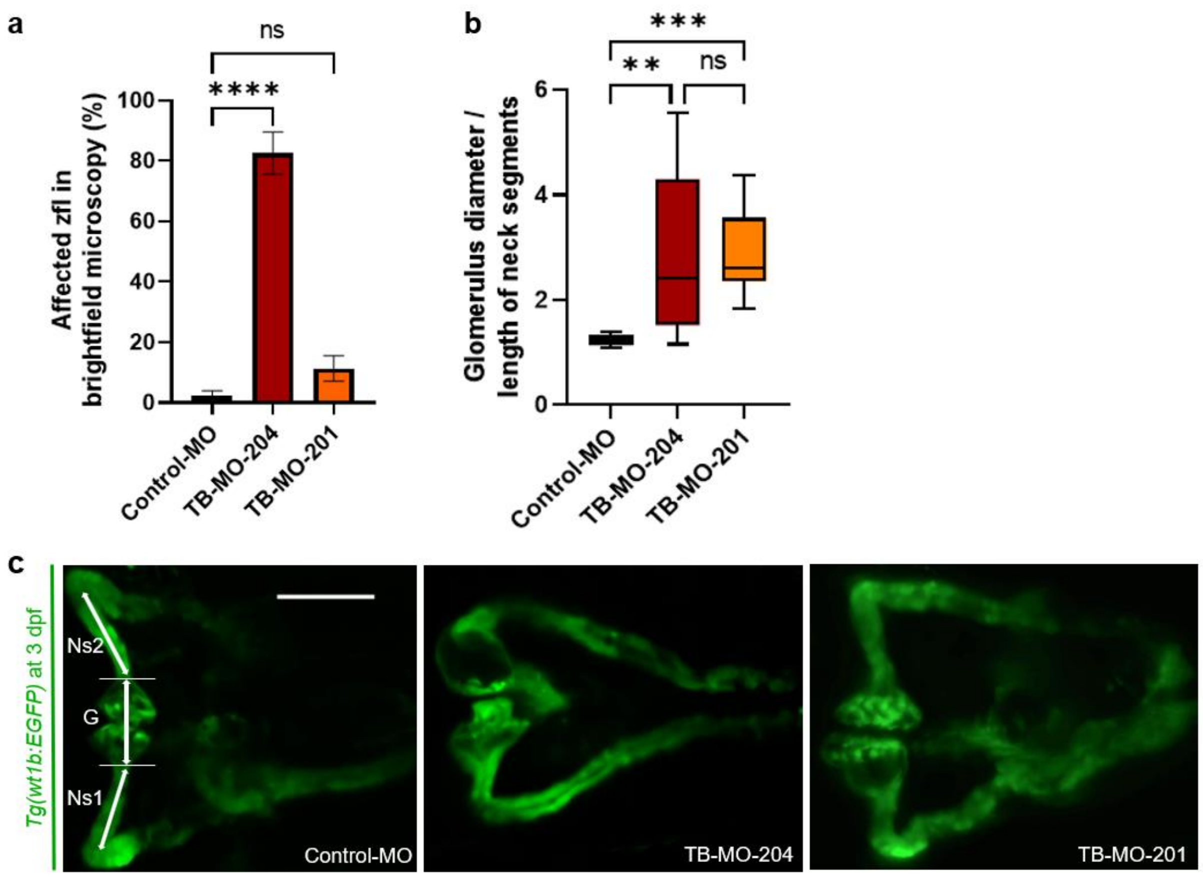

Morpholino® knockdown of Celsr3-201 in zebrafish larvae.

a. – c. Phenotypic evaluation of zebrafish larvae (zfl) injected with Control-Morpholino (Control-MO), zfl injected with MO blocking the *celsr3-204* transcript (TB-MO-204) and zfl injected with MO blocking the *celsr3-201* transcript (TB-MO-201). ** p-value < 0.01, *** p-value < 0.001, **** p-value < 0.0001, *ns* not significant. Two-way ANOVA. Mean with SEM.

a. Percentage of zfl with a warped tail and/or caudal end disruption imaged by brightfield microscopy. We observed a matching phenotype after knockdown (KD) with either TB-MO-204 or TB-MO-201, with a higher phenotypic penetrance of 83 % in TB-MO-204 (Figure 4), compared to 11 % in TB-MO-201 morphants. We therefore assume that a long transcript of *celsr3* does exist in zfl, combining the four partly overlapping sequences from Ensembl database, with the highest agreement to the orthologs in other species. In this case, TB-MO-201 would possibly rather work as a splice-blocking MO with reduced penetrance in phenotypic manifestation. Number (n) of zfl for each injection group Control-MO (n = 157), TB-MO-204 (n = 223) and TB-MO-201 (n = 395). Number of independent experiments N = 3.

b. Box plot showing the size of the glomerulus in relation to the neck segments calculated for each *Tg(wt1b:EGFP)* zfl at three days post fertilization (dpf) (G / ((Ns1 + Ns2) / 2)). Celsr3 MO-KD zfl showed an increase of the glomerular diameter in comparison to the respective length of the neck segments. Control-MO (n = 22), TB-MO-204 (n = 27), TB-MO-201 (n = 35). Number of independent experiments N = 3. **c.** Representative dorsally mounted *Tg(wt1b:EGFP)* zfl at three dpf treated with 1-phenyl 2-thiourea (PTU) and imaged from dorsal to visualize the effect of Celsr3 MO-KD on the development of the pronephros. G: Glomerulus. Ns1: Right neck segment. Ns2: Left neck segment. Scale bar 100 µm.

Supplementary Figure 4

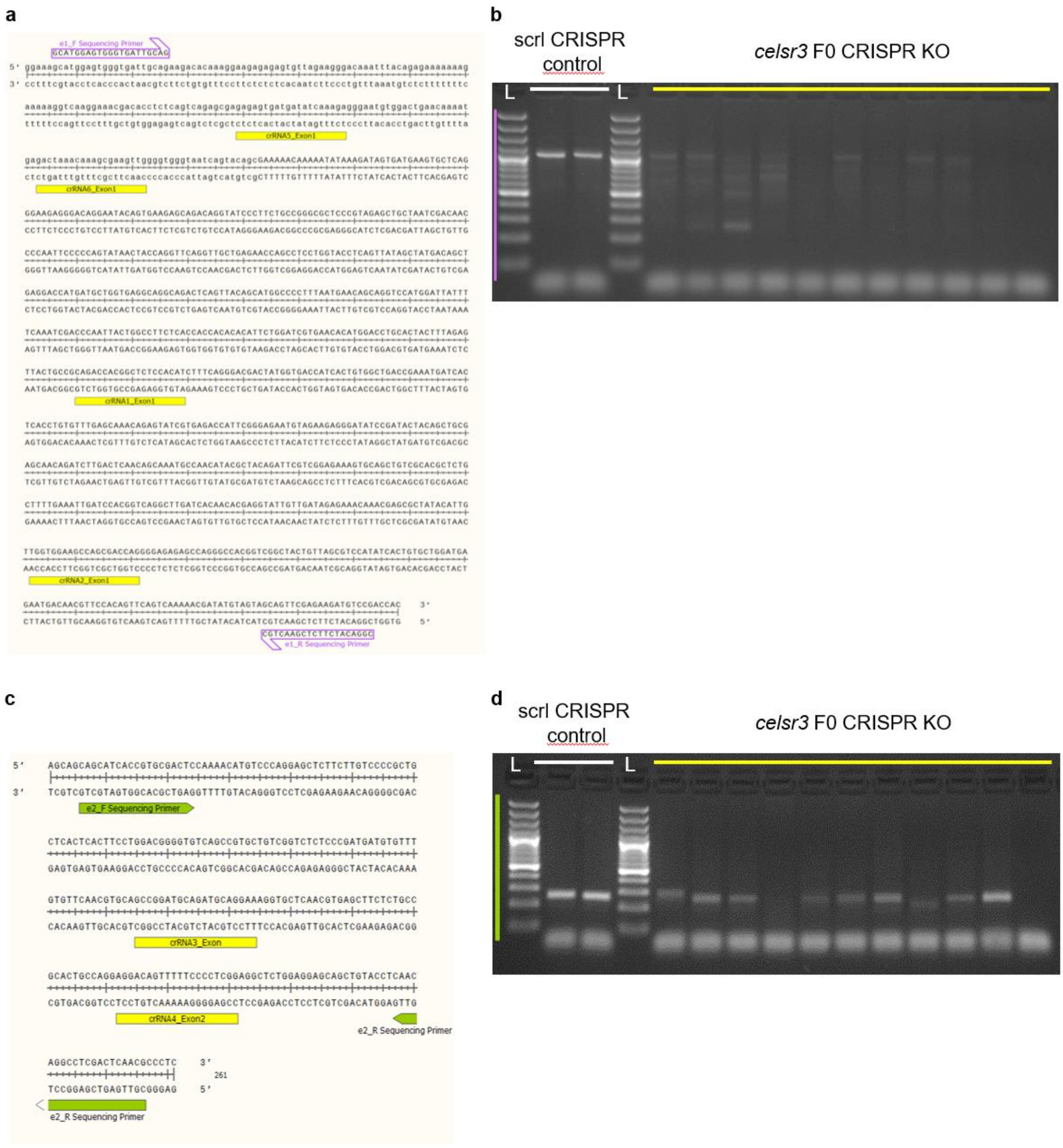

Transient *celsr3* F0 CRISPR-Cas9 knockout in zebrafish larvae.

(a,c) Section of exon 1 (a) and exon 2 (c) *celsr3* gDNA sequence. Binding sites of the crRNA are indicated by yellow bars. Purple arrows indicate the position of the PCR primers. crRNA5_Exon1: GAGAGTGATGATATCAAAGA; crRNA6_Exon1: GACTAAACAAAGCGAAGTTG; crRNA1_Exon1: ATGTGGAGAGCCGTGGTCTG; crRNA2_Exon1: TGGTGGAAGCCAGCGACCAG; crRNA3_Exon2: GCCGGATGCAGATGCAGGAA; crRNA4_Exon2: AGGACAGTTTTTCCCCTCGG; e1_F

Primer: GCATGGAGTGGGTGATTGCAG; e1_R Primer: CGGACATCTTCTCGAACTGC; e2_F Primer: CAGCATCACCGTGCGACTC; e2_R Primer: CGTTGAGTCGAGGCCTGTTG. **(b,d)** Images of PCR gel electrophoresis in *celsr3* F0 CRISPR knockout (KO) or scrambled (scrl) CRISPR control zebrafish larvae (zfl) following the injection of CRISPR-Cas9 system. The e1_F and e1_R primer pair create a DNA fragment of 1018 bp length in scrl CRISPR control - injected zfl (b). The e2_F and e2_R primer pair create a DNA fragment of 251 bp length in scrl CRISPR control - injected zfl (d). In *celsr3* F0 KO zfl, the CRISPR–Cas9 system successfully led to alterations in the genomic sequence, visualized by aberrant fragment lengths (b,d). All gels derived from the same experiment and were processed in parallel. L: DNA ladder.

Supplementary Table 1: Structural modeling of CELSR3 protein variants

Family	Variant	Structural variation	Screenshot Model
**1**	c.1574G>A, p.(Arg525His);<br>c.9299G>C, p.(Gly3100Ala)	The positively charged **Arg525** residue is in the second Cadherin (Cad) domain at the transition to Cad 1. The mutation to histidine takes away the positive charge and could potentially destabilize the region between both Cad domains.<br><br>**Gly3100Ala** is in the C-terminal cytoplasmic domain of CELSR3, which could not be modelled due to missing homologs.	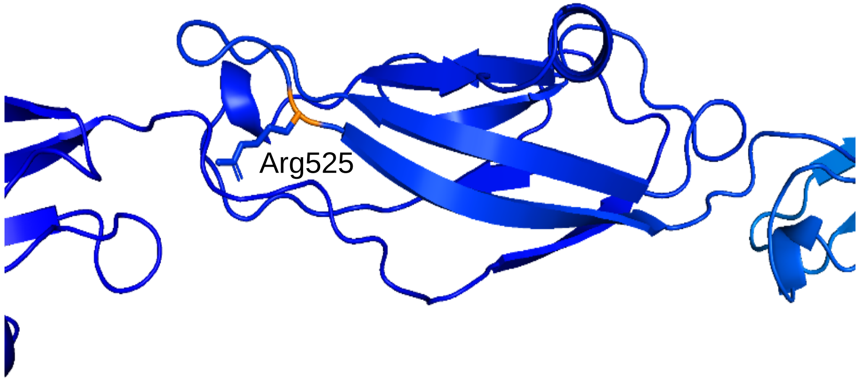

**2**	c.7423C>T, p.(Arg2475Trp); c.8758C>T, p.(Arg2920Trp)	**Arg2475** is in a loop section at the bottom of the GAIN/GPS domain facing the outer side of the membrane leaflet before the Seven-transmembrane (7TM) domain starts. A tryptophan residue instead might change the interaction with the membrane. **Arg2920Trp** is in the C-terminal cytoplasmic domain of CELSR3, which could not be modelled due to missing homologs.	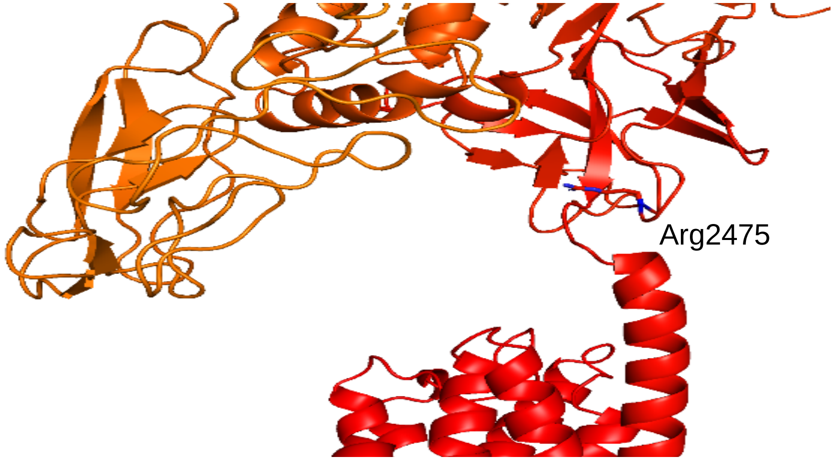
**3**	c.6304G>A, p.(Ala2102Thr)	**Ala2102** is in a loosely defined structured region between the EGF-like region and the GAIN/GPS domain. Its mutation could affect the stability of this region of the structure.	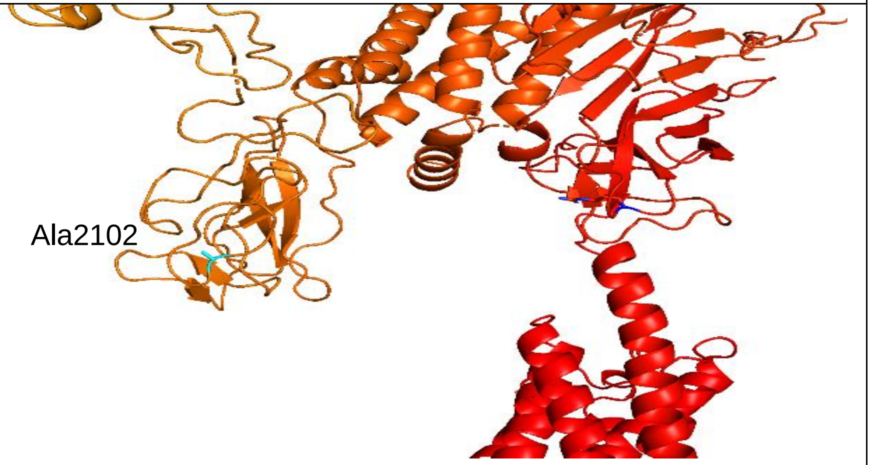

**4**	c.5059C>T, p.(His1687Tyr);<br>c.7075C>T, p.(Pro2359Ser)	**His1687** is in the first of the two Laminin G-like domains. The homologous mutation to tyrosine could affect interaction partners or the stability of this region.<br><br>**Pro2359** is central in the GAIN/GPS domain at the surface of the domain. The region contains many positive charges and a cluster of histidines. The mutation could affect possible interaction partners.	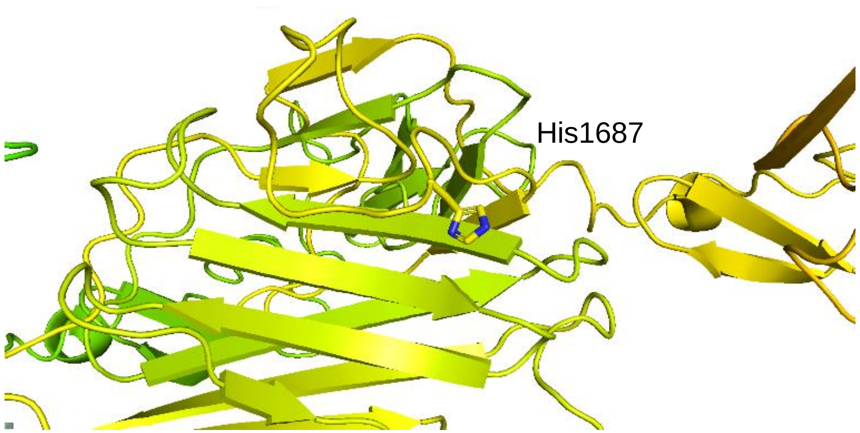<br>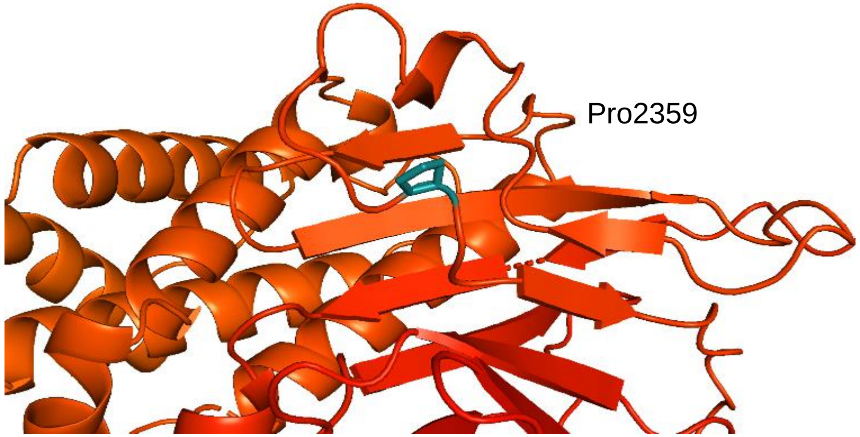

**5**	c.7224_7226del, (p.Ile2409del); c.8480C>A, (p.Thr2827Asn)	The **Ile2409del** mutation introduces a register shift into the side chain up-down sequence of a beta strand in the GAIN/GPS domain, potentially leading to a larger structural disturbance in this area. It is part of a very hydrophobic ISIII$_{2409}$LLV amino acid sequence in this b-strand region.<br><br>**Thr2827** is in the C-terminal cytoplasmic domain of CELSR3, which could not be modelled due to missing homologs.	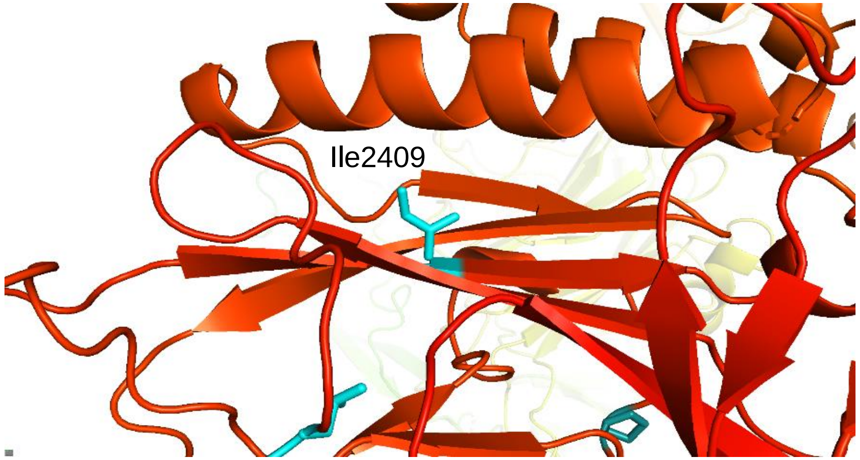
**6**	c.7999G>A, p.(Gly2667Ser)	**Gly2667** is between the fourth and fifth transmembrane helix in an extracellular loop of the TM domain. Its mutation to a polar serine can change the interaction surface of this region.	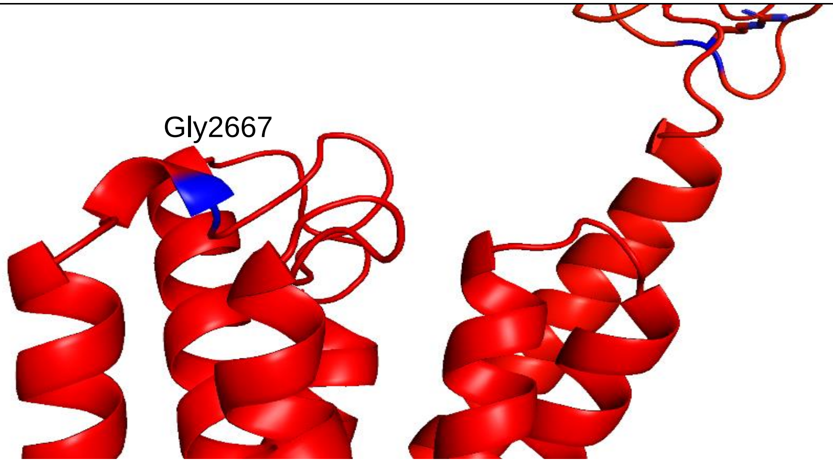

**7**	c.6959T>C, p.(Val2320Ala)	Mutation of **Val2320** to a smaller alanine might induce conformational changes of that loop in the GAIN domain.	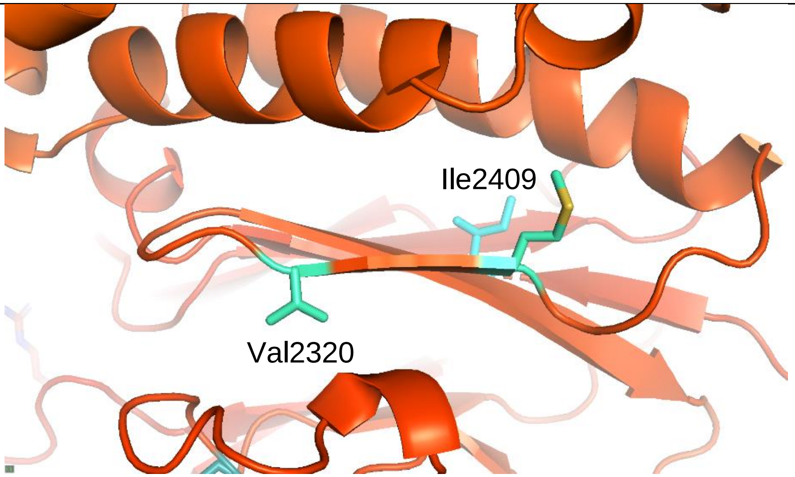
**8**	c.4034C>T, p.(Pro1345Leu)	**Pro1345** is located in a structured region between the 9th Cadherin domain and the 1st EGF-like domain in a very hydrophobic loop. A change to leucine might influence interactions of this domain with other domains or proteins.	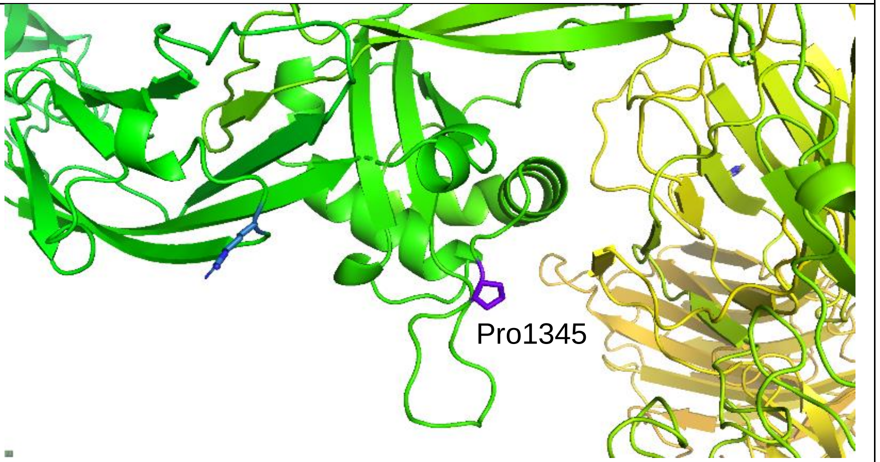

**9**	c.3712C>T, p.(Arg1238Cys);<br>c.7501G>A, p.(Glu2501Lys)	**Arg1238** is exposed on the 9th and last Cad domain. A mutation to cysteine changes the polarity of the domain and might affect possible interaction partners.<br><br>**Glu2501** is located on a terminal b-strand in the GAIN/GPS domain. The mutation from a negative charge to a positive charge can significantly affect the interaction with the membrane or putative interaction partners.	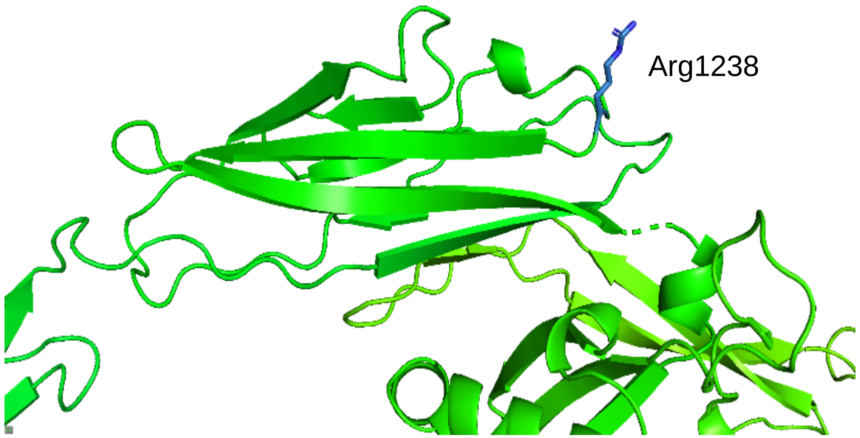 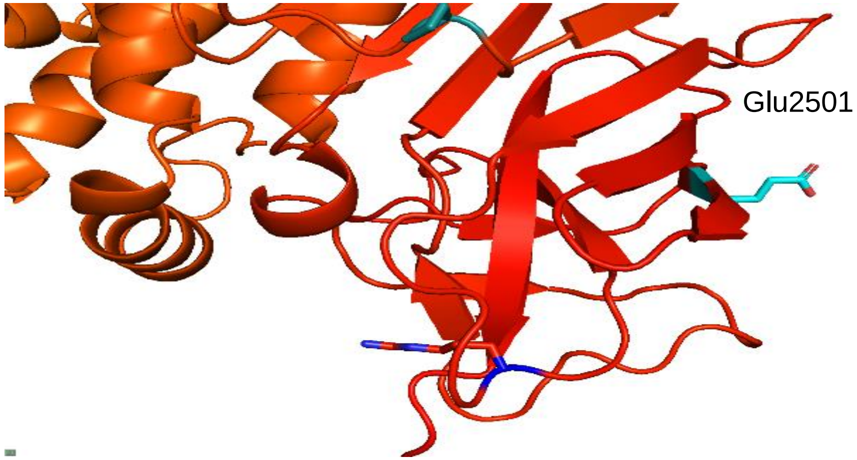

**10**	c.3142C>T, p.(Arg1048Trp)	The large and neutral tryptophan mutation compared to the positively charged **Arg1048**, will certainly change the surface structure of the Cad domain.	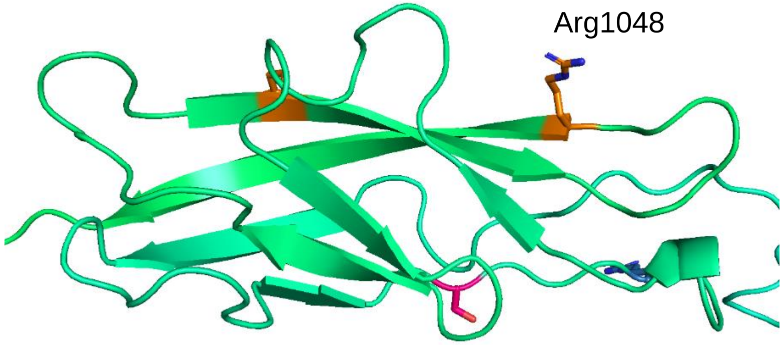
**11**	c.3100G>C, p.(Glu1034Gln)	**Glu1034** is at the surface of the seventh Cad domain and the glutamic acid to glutamine exchange is very conservative. It only takes the negative charge away.	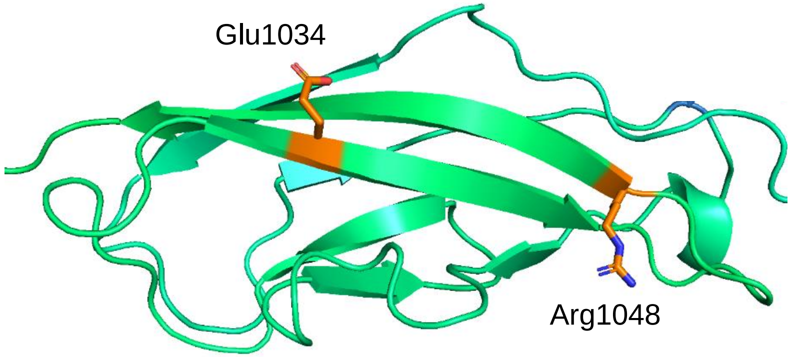

**Supplementary Family 1**	c.1567G>A, p.(Gly523Arg)	**Gly523** (shown here in red) is on the second Cad domain directly preceding Arg525. Its change to the positively charged and highly polar arginine will likely affect the interactions of the Cad domain.	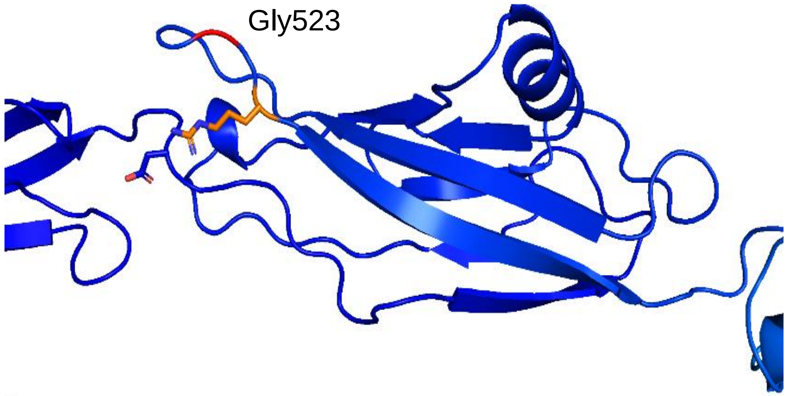

**Supplementary Family 2**	c.7493G>A, p.(Arg2498Gln); c.7886G>A, p.(Arg2629His)	**Arg2498** is another residue in the GAIN/GPS domain close to the TM domain and thus to the outer membrane leaflet. Its exchange to glutamine remains polar but takes the charge away. **Arg2629** is on the cytoplasmic side of the third TM helix. Its mutation to histidine is rather conservative but might influence interactions with phospholipids.	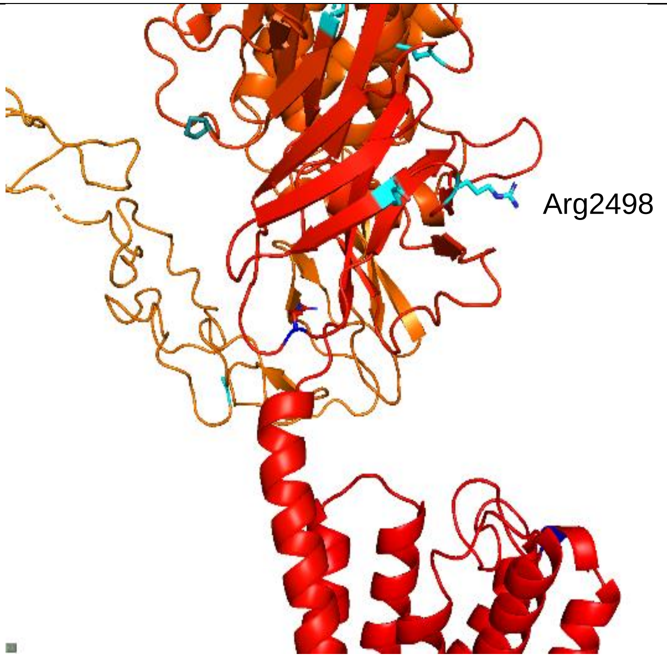

			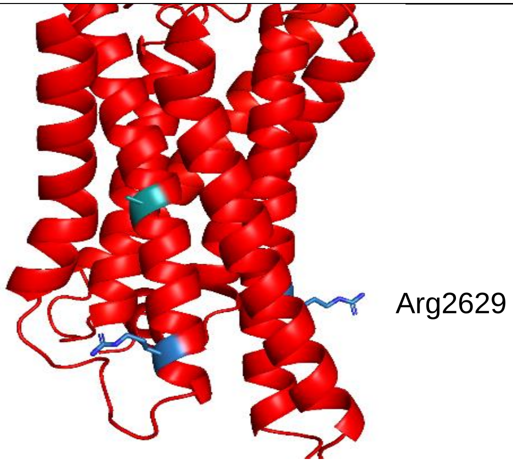
**Supplementary Family 3**	c.8837A>G, p.(Asn2946Ser);<br>c.9170G>A, p.(Gly3057Asp)	**Asn2946** and **Gly3057** are both in the C-terminal cytoplasmic domain of CELSR3, which could not be modelled due to missing homologs.	
